# High-Rate Fingerprinting of Protein Isoforms by Quasi-regulated Enzyme-free Transport Through CytK Nanopores

**DOI:** 10.21203/rs.3.rs-8098579/v1

**Published:** 2026-06-16

**Authors:** Amr Makhamreh, Ali Fallahi, Rik Dhar, Monika Kumari, Siddharth Krishnan, Michele Meseonznik, Luning Yu, Dinara Boyko, Keira Reich-Veillette, Yuhaohua Zheng, Elizabeth A. Libby, Aleksei Aksimentiev, Meni Wanunu

**Affiliations:** 1 Dept. of Bioengineering, Northeastern University, Boston, MA; 2 Dept. of Physics, Northeastern University, Boston, MA; 3 Dept. of Physics, University of Illinois at Urbana-Champaign, Urbana, IL

## Abstract

Nanopores are compelling tools for ultrafast single-molecule protein analysis. Recent investigations into both enzyme-free and enzyme-regulated transport mechanisms suggest that the faster kinetics of enzyme-free transport enable higher throughput, albeit requiring speed control for accurate protein characterization. Here we investigate the signals obtained from chemically unfolded proteins when transported through wild-type cytotoxin K (CytK) in an enzyme-free manner. We designed, expressed, and measured synthetic block isoforms derived from maltose-binding protein that contain repeat sub-sequences as benchmark molecules. Surprisingly, reverse capture of proteins (i.e., from the β-barrel side) proceeds with ~2.5 orders of magnitude higher rate than forward capture, allowing high-rate protein fingerprinting at sub-nM concentrations. We utilize high-voltage reverse capture for rapid analysis of the block isoforms, and found that the ionic current signal shapes, arising from stochastic movements with 4–7 amino-acid-long steps, reproducibly follow the number and order of sequence repeats. We corroborate our results with ionic current signatures obtained from molecular dynamics simulations, exhibiting matching signature shapes. Lastly, we demonstrate an example life-science research use case through rapid nanopore quantification of leaky and overexpressed proteins that contain short electrophoretic tags directly from crude bacterial lysate in under 30 minutes, which allows for rapid and selective quantification of full-length protein expression levels.

## Introduction

Proteins, named from the Greek word *proteios*, meaning ‘of first importance’, serve as the primary drivers of life. Unsurprisingly, proteins are implicated in nearly every disease, making them the central targets for most therapeutics.^1^ They also serve as programmable nanomachines underpinning various biotechnology applications spanning from therapeutic modalities (e.g., monoclonal antibodies,^2,3^ vaccines,^4^ hormones,^5,6^ multifunctional peptides,^5^ cell therapy receptors^7,8^) to bioprocessing and biomanufacturing accelerators (e.g., enzymatic chemical synthesis^9^ and industrial waste processing^10^) to agriculture (e.g., genetically modified crops^11,12^. These valuable use cases have fueled significant advances in the field of proteomics and protein analysis, the last of which has been a revolution in computational approaches for predicting protein structure and activity.^13,14^ Concurrently, there is increasing attention to the challenge of determining the abundance and chemical states (e.g., presence of posttranslational modifications, or PTMs) of proteins and their distinct isoforms, which holds immense promise for advancing our understanding of proteome dynamics and guiding therapeutic development.^15–18^ However, progress remains constrained by the lack of comprehensive and high-throughput tools for protein characterization within complex biological systems.^19^ This limitation is evidenced by the fact that the most employed method for quantifying protein expression to date still relies on surrogate mRNA levels obtained through whole transcriptome analysis.^20^

Mass-spectrometry (MS)-based proteomics typically involves fragmentation of proteins into short peptides, their detection, and then ‘bottom-up’ reverse assembly to provide proteome-wide quantification. MS methods to date cannot obtain full proteome coverage nor detect novel proteoforms effectively.^21–25^ Although transcriptomic data and the identification of peptides containing a splice junction can suggest the presence of a proteoform, accurate characterization of its expression levels and combinatorial PTM profile requires analysis at the full-length protein level.^23,26,27^ Methods that offer reliable detection of intact proteoforms are needed since the expression and relative abundance of protein isoforms directly impact a wide range of cellular functions and disease phenotypes.^28–31^ Often, the challenge of differentiating proteoforms does not require single amino acid resolution since the majority of known proteoforms are significantly different in sequence length, with >40% varying by more than 100 amino acids.^32^

Nanopores can in principle provide proteoform-aware detection, as every region of a protein molecule can modulate the measured ionic current signal. Direct nanopore nucleic acid sequencing is distinguished from other legacy sequencing methods due to the ability to sequence native molecules, which circumvents replication-based errors and allows for the detection of chemical base modifications. This advantage has motivated nearly three decades of work since the first demonstrations of nucleic acid transport through biological nanopores,^33^ which have led to the realization of DNA^34^ and RNA^35^ sequencing. Since proteins cannot be universally amplified, nanopores are attractive for high-throughput, single-molecule, and full-length protein analysis.

Nanopores can also be used in a “fragment detection” mode to analyze properties of peptides (e.g., molecular weight) after enzymatic digestion, resolving known isoforms^36,37^ and identifying chemical modifications histone tails.^38^ However, irrespective of the resolution, this approach commonly requires a consensus of many single-molecule observations for characterizing an isolated protein sample. Although, it has recently been demonstrated that accurate peptide classification can be achieved at the single-event level.^39^ Full-length protein fingerprinting with solid-state nanopores was recently shown by counting current-enhancement spikes from cysteine-linked oligonucleotides.^40^ While promising, the classification performance of this technique is inherently limited by the labeling efficiency of the target residue, where even successful labeling does not guarantee that all its representative spikes are detectable. Moreover, the inability to couple signal detection with positional context makes it difficult to distinguish proteins that share a similar number of a target residue.

Controlled protein transport through a nanopore requires their unfolding, terminal threading, and a unidirectional process that overcomes the non-uniform charge distribution along the protein’s contour. Two main strategies have been developed to overcome these challenges: 1. enzyme-driven translocation of full-length proteins^41–43^ or DNA-linked protein fragments,^44,45^ and 2. enzyme-free, denaturant-assisted protein translocation.^46–55^ We and others have demonstrated enzyme-free translocation of full-length proteins through nanopores by combining chemical denaturants, such as guanidinium chloride (GdmCl) or urea, to unfold proteins and transport them using electroosmotic flow (EOF). Sustained EOF through the pore counteracts bi-directional electrophoretic forces (EPFs) induced by the non-uniform charge distribution along the protein analyte contour.^50–52,55^ Induction of EOF can be achieved by engineering the nanopore surface charge,^56,57^ but it can also be induced by Gdm^+^ ion adsorption to nanopore surfaces.^50,58^ Previously, we used a α-hemolysin pore to obtain fingerprint signatures from different proteins, showing that machine-learning (ML) can accurately discriminate different proteins based on their translocation signal patterns.^50^ However, the ~10 μs per amino acid speed of protein transport in that system approaches the practical limit of current sampling afforded by most nanopore amplifiers. Further reduction of the protein transport speed would enable extraction of more signal-level features, essential for accurate proteoform fingerprinting.

In this work, we investigate the ability of wild-type cytotoxin K (CytK) nanopores to resolve the signals obtained from full-length synthetic proteins derived from maltose binding protein (MBP) sub-sequences, which we refer to as protein block isoforms (PBI). Specifically, we evaluated the capture rates, transport speeds, and ionic current signatures of MBP and four engineered PBIs, containing blocks of the parent MBP sequence that repeat in various permutations. First, we show that protein transport through CytK is ~6 times slower than α-hemolysin under the same experimental conditions. Second, we find that the protein capture with reverse transport (i.e., from *trans*-to-*cis*) provides >300 times more likely compared to forward transport (i.e., from *cis*-to-*trans*), which we conjecture stems from two effects, namely the high localized electric field near the *trans* side opening (data from MD simulations), and interactions of the denatured protein with the lipid membrane. Third, we show that the signatures produced by the isoforms are unique, exhibiting a sequence-dependent repetition. Fourth, we demonstrate quantification of PBIs from mixtures at sub-nM levels, including a 5-way proteoform mixture. Finally, we harnessed our high-sensitivity platform to demonstrate rapid and selective detection of leaky and overexpressed proteins directly from *E. coli* crude lysates. We demonstrate full-length expressed protein quantification in ~30 minutes post-lysis, which reduces detection time tremendously when compared against Western blot analysis, for example. Our work presents a viable path towards high-throughput characterization of mixtures of similar proteins with pM sensitivity using nanopores.

## Results

### Slow and high-capture transport of proteins through a CytK nanopore

**Figure 1.**
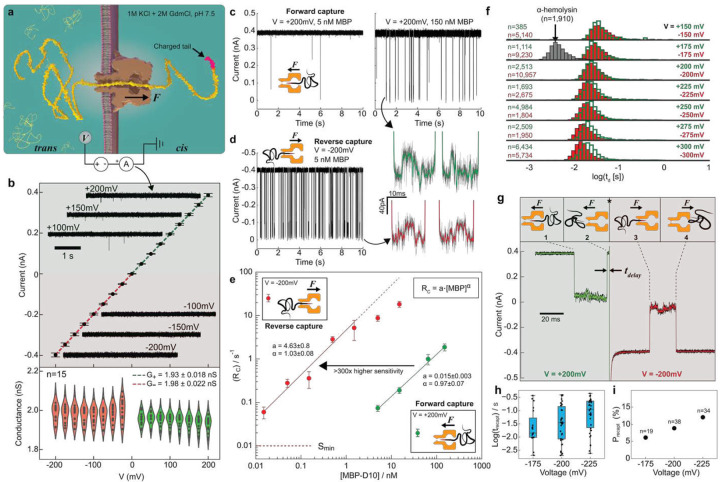
Reverse capture and transport of proteins through CytK nanopores. **a,** Cross-section view depicting reverse translocation (*trans-to-cis*) of a GdmCl-unfolded protein analyte with a negatively charged tail at V<0 through a wild-type Cytotoxin-K (CytK-WT) nanopore inserted into a DPhPC bilayer membrane. Protein transport through the β-barrel of CytK is initiated by the electrophoretic capture of the terminal charged tail and electroosmotic flow (EOF) drives protein transport through the pore. **b,** Average (n=15) current-voltage (IV) trend of a CytK nanopore in 1M KCl, 2M GdmCl, 10mM Tris, pH 7.5 (top). Voltage values refer to V applied to the *trans* chamber. Conductance versus voltage of CytK shown as violin plots (bottom). **c,** Current versus time traces showing forward translocation of MBP through CytK at +200mV at indicated MBP concentrations. Example of forward translocation events of MBP with a 25 kHz low-pass Bessel filter (black) overlaid with a 1.5 kHz low-pass Bessel filter (bottom right, green). **d,** Current versus time traces showing reverse translocations of MBP through CytK at −200mV at a 5 nM concentration. Example of similar *reverse* translocation events of MBP through CytK with a 50 kHz low-pass Bessel filter (black) overlaid with a 1.5 kHz low-pass Bessel filter (right, red). **e,** Capture rate versus concentration of MBP through CytK via reverse translocation at −200 mV (red) and forward translocation at +200 mV (green) with power law fit for each shown. False positive event rate observed with CytK at −200mV with 0 nM MBP (control), i.e., minimum sensitivity (S_min_), is shown (red dashed line). **f,** Log-scaled dwell-time (t_d_) distributions for MBP forward (green, hollow) and reverse (red, filled) translocations through CytK at different voltages. The t_d_ distribution of MBP observed with alpha-hemolysin at +175 mV (gray) is shown for comparison. **g,** Trace showing a recapturing event of MBP with CytK, where 2 ms after forward translocation of MBP through CytK at +200mV (green), a trigger switched the voltage to −200mV, allowing for the same MBP protein to be recaptured and subsequently undergo reverse translocation (red). **h,** Distribution of MBP recapture time versus voltage shown as box and whiskers plots. **i,** Probability (%) of MBP recapture versus forward/reverse voltage.

Our experimental setup consists of a vertical two-chamber fluidic cell contacted by a ~100 μm diameter planar lipid membrane, through which a single CytK channel spontaneously inserts (see Fig 1a and **Methods**). Conventionally, we define the *cis* chamber as the side that faces the CytK vestibule, and to which the ground electrode is connected (all indicated V values in the manuscript refer to the *trans* chamber voltage). We investigated reverse translocation of full-length denatured protein molecules (yellow) that contain a polyanionic D_10_ tail (shown in red), which facilitates threading^50^. For all experiments in this study, both the *cis* and *trans* chambers were filled with 500 μL electrolyte buffer containing 1M KCl, 2M GdmCl, 10 mM Tris, pH 7.5. We characterized the conductance and stability of CytK in multiple experiments (n = 15), and current-voltage curves (Fig. 1b, top) show reproducibly linear trends at both positive (green) and negative (red) voltages with stable baseline currents in both directions (example traces from a single CytK channel shown as insets). However, we observed a slight asymmetry in the conductance, where at positive and negative voltages, the average conductance was <G_+_> = 1.93–0.02 nS (green) and <G_-_> =1.98–0.02 nS (red), respectively (Fig. 1b, bottom), similar to α-hemolysin under the same buffer conditions^50^. Surprisingly, the baseline is stable for both voltage polarities, with minimal gating even at high voltage magnitudes. Thus, at −200mV, the observed gating frequency was 0.02 s^−1^ (Supplementary Fig. 1), where gating is defined as a long-lived pore current reduction that requires a voltage reversal to reopen the pore.

The stability observed in the open pore baseline of CytK at positive and negative polarities enabled our comparison of unfolded protein capture and transport in both the forward and reverse directions. First, as expected, we observe an increase in the frequency of transport events with forward transport of MBP through CytK when the [MBP]_*cis*_ concentration is increased from 5 nM to 150 nM (Fig. 1c). The concentration-normalized capture rate of MBP through CytK (0.013–0.002 nM^−1^s^−1^) is in agreement with our prior study^50^ using α-hemolysin pores at +200 mV (0.011 nM^−1^s^−1^). Under the same conditions, we surprisingly observe a much higher capture rate for reverse MBP transport at [MBP]_*trans*_ = 5 nM at −200 mV (Fig. 1d) compared to forward transport (1.75–0.27 nM^−1^s^−1^ vs. 0.013–0.002 nM^−1^s^−1^, respectively). At higher concentrations ([MBP]_*trans*_ > 10 nM), we find that the pore is persistently in a blocked state (Supplementary Fig. 2). While a previous report^54^ proposed that “irreversible blockades” observed from the *trans* side are due to peptide/lipid interactions, we find that the reason for the pore being in a blocked state is due to high protein capture in reverse transport mode (Supplementary Fig. 2). The discrepancy in forward and reverse transport appears to be limited to the capture rate, as the translocation events show very similar event shapes (Fig. 1c, green traces and Fig. 1d, red traces).

To quantify the enhanced capture efficiency observed with reverse transport through CytK, we measured the concentration-dependent forward and reverse capture rates of MBP under the same voltage (|V| = 200 mV) (Fig. 1e). As shown in the log-log plot of mean capture rates vs. MBP concentration in the forward (green) and reverse (red) directions (Fig. 1e), both directions fit power law functions with near-linear behavior (α ~ 1). From the amplitude parameters of the power law fits, we found that reverse capture of MBP is >300x more efficient than forward capture, a large sensitivity enhancement. Based on our capture vs. concentration data, our curve intersects the false-positive (FP) event rate at ~2.5 pM (Fig. 1e, dashed red line), for which we observe ~1 false event per 50 seconds of recording (Supplementary Fig. 1a). Nonetheless, since FP events have different signal characteristics (amplitude and duration) than true protein transport events (see Supplementary Fig. 1b), 2.5 pM does not represent the absolute lower limit of detection. Additionally, for MBP-WT which lacks the D_10_ tail, reverse translocation was not observed at −200 mV and −300 mV (Supplementary Fig. 3), consistent with prior data with α-hemolysin.^50^

**Extended Data Figure 1.**
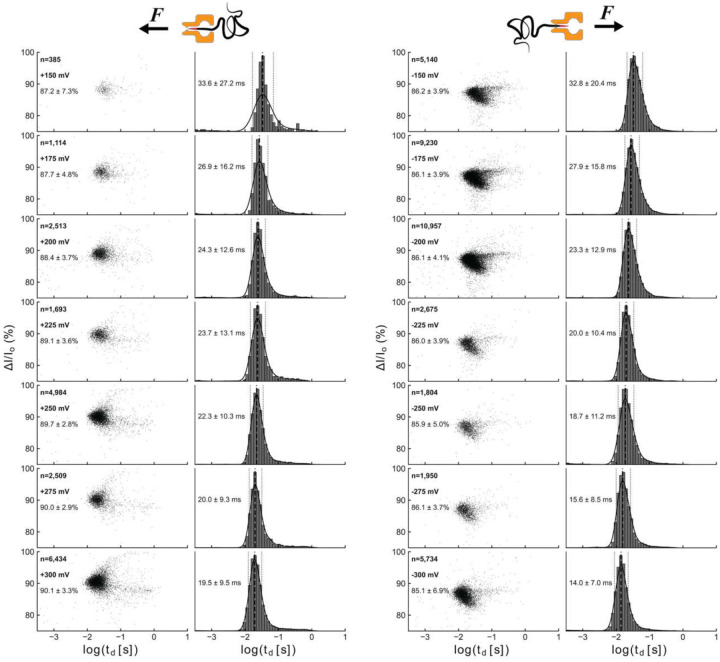
Fractional blockade versus dwell time of forward and reverse translocation of MBP through CytK. Shown on the left are scatter plots of the fractional blockade (ΔI/Io) versus log(t_d_) of MBP translocation events through the *cis* side (left) and *trans* side of CytK (right). For each scatter, the voltage, sample size, and μ±σ are provided. Shown on the right are the distributions for the log(t_d_) of MBP translocation events, with each dwell time histogram corresponding to the scatter plot on the left. Dwell time histograms are fit with a Gaussian kernel-density estimate (KDE), where the full width half maximum (FWHM) of the KDE fit are shown as the thin dashed lines and the KDE mode of each distribution is shown as the thick black line overlaid on the histogram. The KDE mode–FWHM at each voltage is provided.

As for the fractional current blockade statistics, we found ΔI/I_o_ levels of 88–4.2% and 86–3.9% for forward and reverse translocations of MBP, respectively (Extended Data Fig. 1), similar to the 87.5% observed with α-hemolysin at +175 mV. Upon inspection of the current signals obtained during reverse transport, we hypothesize that the slight reduction in the mean fractional blockade in the reverse transport of MBP is due to a population of events that (1) exhibit the lower blockade state for a considerable portion of the transport time and/or (2) have a shallow blockade level right before MBP exits the pore and the current returns to the open pore state (Supplementary Fig. 4).

To investigate the transport dynamics of forward vs. reverse transport, in Fig. 1f we show transport time, or dwell time (t_d_), distributions at various applied voltages. We find that mean t_d_ values decrease monotonically with voltage for both forward and reverse translocation up to −300 mV, which supports a translocation mechanism (see also Extended Data Fig. 1 and Methods). Reverse transport of MBP had a slightly sharper decrease in t_d_ and tighter distribution as a function of voltage when compared to forward transport, indicating that reverse translocation provides smoother protein transport dynamics (Extended Data Fig. 1). Moreover, the slower transport dynamics of MBP through CytK enables high-voltage (−300 mV) recordings, which improves the signal-to-noise of our measurements. The ability to translocate proteins at higher voltages contrasts with a recent report using a CytK-4D mutant,^54^ for which most events were long-lived blockades at voltages greater than 120 mV, indicative of protein clogging. For comparison, in Fig. 1f we superimpose a dwell time distribution for forward transport through α-hemolysin at +175 mV (grey). With <t_d_> ~5 ms for α-hemolysin and 26.9 ms and 27.9 ms for forward and reverse transport through CytK at |V| = 175 mV, respectively, transport through CytK is ~6 times slower than through α-hemolysin, despite their similar overall pore structures.^54^

**Extended Data Figure 2.**
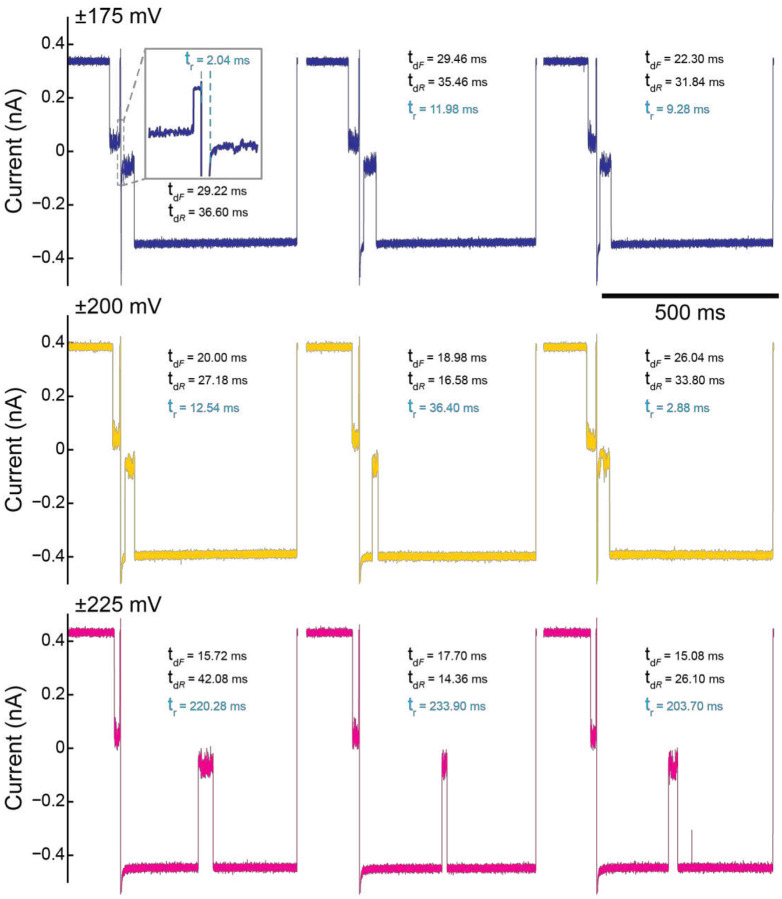
Example traces of MBP recapture events at different voltages. Traces showing event where the same MBP molecule was successfully recaptured through the *trans* side of CytK (reverse translocation) at 175 mV (top, blue), 200 mV (middle, gold), and 225 mV (bottom, pink). The event dwell times for forward transport, reverse transport, and the recapture dwell time for each event are denoted as t_dF_, t_dR_, and t_r_ (cyan), respectively. Recapture of MBP was achieved by applying a negative bias for 500 ms after forward translocation of MBP was completed. A 2 ms delay was set prior to triggering the negative bias needed for attempting the recapture of the same molecule.

The similar forward and reverse protein transport dynamics suggests that the same molecule could be observed at least twice, once forward and once in reverse upon its recapture, as has been demonstrated in past works for DNA.^59–62^ In Fig. 1g, we show a current trace for a recaptured molecule after forward transport. For this acquisition we employed a threshold-triggered voltage waveform where upon exit of a molecule from the pore in forward transport mode, the voltage is reversed with a delay time of δt = 2 ms, and the reverse voltage is held for 500 ms to “hunt” for a recapture event (see Methods). In the main trace shown, the forward and reverse events had similar dwell times and shapes, although this was not always the case. We summarize the recapture time statistics (Fig. 1h) and the recapture probabilities (Fig. 1i) at three different voltages, −175 mV, −200 mV, and −225 mV (see also Extended Data Fig. 2). Two noteworthy observations made in these experiments: 1. at higher forward voltages, mean recapture times were longer, since molecules diffuse farther away from the pore (Fig. 1h, Extended Data Fig. 2), and 2. the probability of recapture increases with the recapture voltage.

### Molecular dynamics simulations of protein transport through CytK.

**Figure 2:**
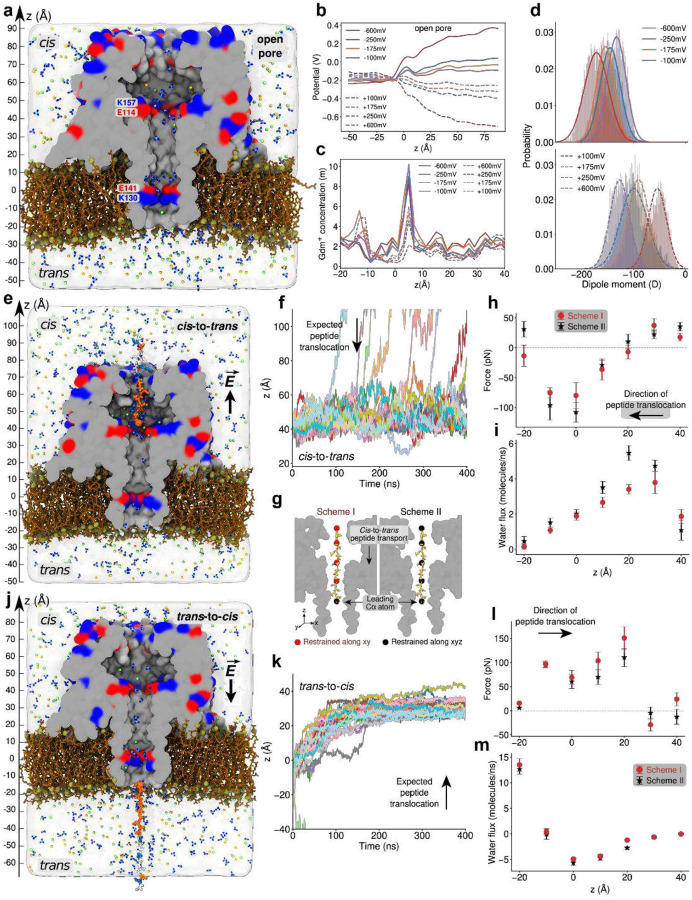
MD simulations of CytK systems. **a,** Cut-away view of a CytK nanopore (gray) embedded in a lipid membrane (ochre) and immersed in 1M KCl / 2 M GdmCl electrolyte (semi-transparent surface). Cationic and anionic amino acids are shown in blue and red, respectively, K^+^ and Cl^−^ ions as yellow and green spheres, and Gdm^+^ as cyan and blue bonds. The image shows representative configuration after 270 ns simulation at −250 mV. **b,** Average electrostatic potential along the pore axis. **c,** Distribution of Gdm^+^ ions along the nanopore axis. The Gdm^+^ concentration was computed using 12 Å radius cylindrical bins stacked in 2 Å intervals along the pore axis. **d,** Dipole moment of Lys_130_ and Glu_141_ residues along the pore axis at specified bias conditions sampled every 40 ps during the respective MD trajectory. **e,** Cut-away view of the system used for the simulations of forward transport. The analyte peptide is color-coded according to amino acid charge: anionic in orange, cationic in blue, and uncharged in silver. **f,** Simulated displacement of the analyte peptide under a +1.2 V bias characterized by the z-coordinate of its leading residue Cα atom (orange sphere in panel **e**). Traces from 24 replica simulations are shown in different colors. **g,** Schematics of the two methods used to measure the effective force on the peptide in forward transport. **h,** Effective force experienced by the peptide and **i,** average water flux through the nanopore for seven placements of the peptide within a CytK nanopore in forward transport. For each data point, the error bar shows the standard error of mean computed by splitting the three 210 ns long MD trajectories into 30 ns fragments. **j-m,** Same as in panels **e** but for reverse transport.

To identify the molecular mechanisms responsible for the differences in the capture efficiencies between forward and reverse transport, we built an all-atom model of CytK pore embedded in a lipid membrane and submerged in a 1M KCl / 2M GdmCl electrolyte mixture (Fig. 2a). Upon equilibration, the system was simulated in 1M KCl, 2M GdmCl under several transmembrane voltages to determine the IV dependence of the pore and the ion selectivity of the current (Supplementary Fig. 5a-d). The resulting IV curve of CytK was linear, with the average conductance of 1.935 – 0.020 nS, consistent with the 1.9 nS value seen in experiment. About 65% of the total ionic current comprises Cl^−^ ions, regardless of the magnitude and direction of the voltage. Consistent with the observed ion selectivity, a transmembrane voltage produced an EOF in the direction opposite that of the transmembrane bias (Supplementary Fig. 5e, f). At negative voltage the EOF flux was seen to gradually increase with the voltage magnitude, while at positive voltage the EOF flux was nearly constant within the error bars of our computational experiments.

Computational analysis of our open pore trajectories revealed the distribution of the electrostatic potential within the open CytK pore (Fig. 2b). The electrostatic profile shapes changed differently with voltage polarity. Most notably, a local maximum of the potential was observed near residues Lys130 and Glu141 at negative voltages, which in our experiments produces reverse protein translocations. Two factors are responsible for the emergence of the peak. First, we observed pronounced accumulation of the Gdm+ ions upstream from the rings of Lys130 and Glu141 residues, (Fig. 2c, Supplementary Fig. 5g) at negative voltages, which can also be seen in a microscopic image of the simulated system (Fig. 2a). The local enhancement of Gdm+ concentration was seen to disappear as the voltage changed from −600 to +600 mV (Fig. 2c). Simultaneously, we observed a major decrease in the dipole moment magnitude of Lys130 and Glu141 residues (Fig. 2d) along the pore axis. Thus, at negative biases, external electric field separates the positive charge of the Lys130 ring from the negative charge of the Glu141 ring, which promotes binding of the incoming Gdm+ ions upstream from the Glu141 ring, and thus increases the local density of Gdm+ ions. In contrast, a positive bias promotes intermingling of the Lys130 and Glu141 side chains, reducing their dipole moment and their ability to retain Gdm+ ions. The slight yet discernable motion of the Lys130 and Glu141 side chains (compare Fig. 2e and Fig. 2j) was found to alter the nanopore cross-section (Supplementary Fig. 5h, i), which may be responsible for a complex dependence of the EOF flux on the voltage.

To directly observe the effect of the bias polarity on protein capture, we built two simulation systems (Fig. 2e,j) each containing the first twenty residues of a D_10_-modified MBP protein. The peptides were initially oriented to reproduce the forward and the reverse capture geometries, which we experimentally observed at positive and negative voltages, respectively. To observe statistically significant number of peptide capture events within the time scale of our simulations, 24 replicas of each system were simulated in parallel at +/−1.2V voltage, restraining the motion of the 1^st^, 5^th^, 10^th^, 15^th^ and 20^th^ Cα atoms of the peptide along the pore axis (see Methods). In the case of the forward transport system, the peptide was observed to transiently approach the stem of CytK from its vestibule but failed to enter in all 24 simulations (Fig. 2f). On the contrary, twenty-three out of 24 peptides were captured by the CytK stem in the reverse transport configuration (Fig. 2k). SI Movie 1 and 2 illustrate typical simulation trajectories. Thus, our computational assay reproduced the experimentally determined asymmetry of peptide capture.

To quantitatively assess the forces driving the peptide translocation and to elucidate the physical factors responsible for the observed behavior, we measured the effective force applied to the peptide by restraining its selected atoms to pre-determined positions within the nanopore (Fig. 2g). Our data show that, as the peptide enters the nanopore in the forward transport orientation, it experiences an effective force opposing its motion until its first residue is able to move half-way through the CytK stem (Fig. 2h). Once it has reached the half-way point, the effective force changes its sign, acting in the direction of the transport. In contrast, a peptide entering CytK in the reverse transport orientation experiences an effective force that acts along the direction of the transport almost all the way through the stem (Fig. 2l). Thus, our effective force simulations fully corroborate the results of peptide capture simulations.

Further analysis details the action of water flow in our effective force and capture simulations: In forward transport, the water flux is strong and directs out from the channel, pushing the peptide away from the entrance into the stem (Fig. 2i). The water flux magnitude reduces as the peptide enters deeper into the channel, but the direction remains such to oppose transmembrane transport. This effect is explained by the charge of the D_10_ tag that alters the selectivity of the ionic current (Supplementary Fig. 6a-e) and reverses the sticky action of Gdm^+^ ions.^58^ In reverse transport, the water flux is initially directed to promote the capture of the peptide by the nanopore (Fig. 2m). As the peptide enters the nanopore, the flux changes direction, pushing the peptide back toward the trans side, which occurs because the D_10_ tag changes the ion selectivity (Supplementary Fig. 6f-l). However, the force of the EOF is, apparently, not sufficient to push the peptide back because the D_10_ chain interacts favorably with Gdm+ ions accumulated upstream from the ring of negatively charged Glu141 residues. Altogether, the dipole moment gate of the CytK channel (residues Lys130 and Glu141) that induces preferential accumulation of Gdm+ near the gate in the reverse translocation direction and the very charge of the D_10_ tag are responsible for the pronounced directional asymmetry of the peptide capture

**Extended Data Figure 3.**
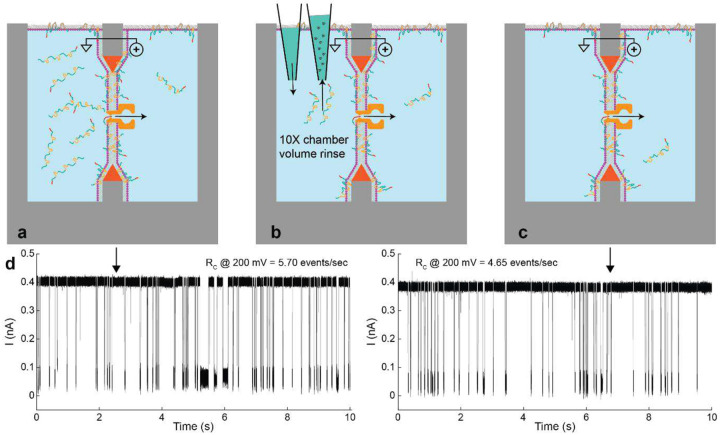
Protein capture rate conserved with reverse transport after their removal from the *trans* chamber due to membrane binding. **a,** Scheme showing reverse translocation of denatured proteins through the β-barrel of CytK from the *trans* chamber. Protein analytes are adsorbed to the lipid membrane, increasing their concentration near the pore opening and their propensity to be captured. **b,** Removal of protein analytes in the *trans* chamber via buffer exchange using syringe pumps. One pump aspirates the working solution containing the analyte from the *trans*. Simultaneously, the second pump perfuses an equal volume of solution to replace the aspirated volume. **c,** After dilution of the analyte in the bulk solution, reverse protein translocations persist due to denatured proteins remaining bound to the side of the membrane facing the *trans* chamber. **d,** Continuous current versus time traces showing reverse translocation of an MBP-derived protein isoform (P3) at 200 mV before (left, corresponding to panel **a**) and after (right, corresponding to panel **c**) 10x *trans* chamber wash. The mean capture rate is annotated at the top of each trace.

A second factor that contributes to the capture rate enhancement is the non-specific adsorption of denatured protein analytes on the membrane surface. This accumulation, or pre-concentration of analytes on the membrane, can lead to higher local concentrations near the pore than bulk analyte concentrations. To investigate this we compared the capture rates of protein transport through the β-barrel of CytK before and after the removal of protein from the chamber (Extended Data Fig. 3a–c) which was carried out with simultaneous perfusion and aspiration of fresh buffer. Despite removing the protein suspended in the bulk solution, we observed a similar capture rate to the one prior to flushing (Extended Data Fig. 3d), providing evidence that proteins in their denatured state are binding and concentrating onto the membrane. These interactions magnify the role of pore geometry on capture – the pore’s β-barrel is closer to the membrane headgroups (higher reverse capture rates) than the top of the vestibule, which is farther away from the membrane (lower forward capture rates).

### Design and translocation of protein block isoforms

**Figure 3.**
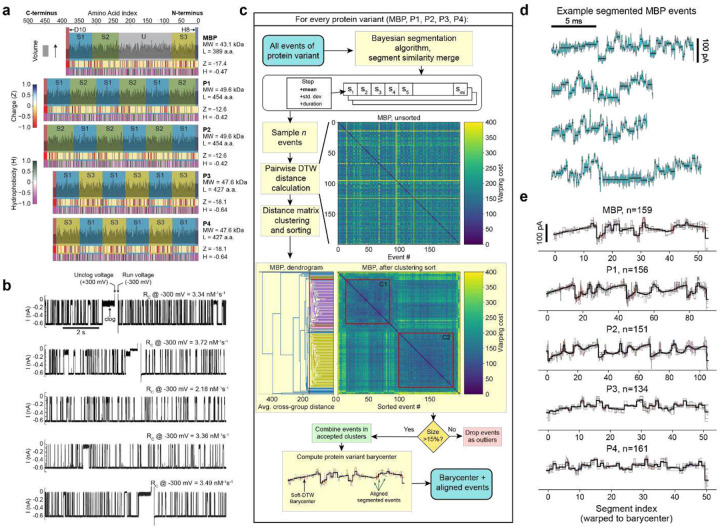
Signal segmentation and average shape extraction from translocation events of protein variants. **a,** Graphical representations of the relative amino acid (aa) volumes (top, gray), overall protein charges at pH 7.5 (Z, middle), and hydrophobicity (H, bottom) for MBP and all protein block isoforms (PBI) used in this study in their unfolded (solvent accessible) state oriented from C to N terminus. The locations of each aa sequence block from MBP used to make the PBIs, i.e., S1 (cyan), S2 (green), S3 (yellow), the poly-aspartic tail (red), and the histidine tag (dark blue) are shown as the colored segments overlayed on top of the aa volume plot. To the right of the graphical representation are bulk properties for each analyte, which are the aa length (L), molecular weight (MW), net charge at pH 7.5 (Z), and hydrophobicity score (H). **b,** Example of a 10 second continuous trace showing reverse translocation of each analyte through a CytK nanopore in 1M KCl, 2M GdmCl, 10mM Tris, pH 7.5 at −300 mV. Traces were digitally low-pass filtered at 25 kHz. Clogging events and subsequent voltage reversals used to eject clogged analyte are shown. The capture rate normalized to concentration at −300 mV for each protein analyte is annotated at the top of each trace. **c,** Signal processing pipeline to convert raw event currents for every protein variant to a barycenter and set of aligned events of that variant. This pipeline is separately applied to datasets of each variant (MBP and P1-P4); MBP data is shown as an example. Upon segmentation, means of steps are used for downstream processing. A random sample of *n*=200 events from a variant dataset is taken for pairwise DTW distance matrix calculation, followed by hierarchical clustering. Clusters are formed below 0.85th quantile of tree branching nodes, and the clusters containing less than 15% of the starting *n* are discarded as outliers. The remaining events (from either one or two clusters for different analytes) are pooled together and used to compute the barycenter of each protein analyte. Through Soft-DTW barycenter averaging (smoothing factor *γ* derived from median number of steps in events), an average shape of segmented events is extracted. The events were then realigned to the barycenter through pairwise Soft-DTW (*γ*=50). **d,** example current traces of translocation events of MBP with Bayesian segmentation output overlaid (black lines and shaded regions indicating mean and – S.D. of each step, respectively steps). Every event is segmented into a different number of steps. **e,** The shapes of the barycenters of each protein variant (black) along with 7 randomly picked events (colored lines). Only the step means are shown for clarity. Here, *n* indicates the number of events that were collected in clustering and pooling for computation of each barycenter.

Given the ~6-fold slower protein transport dynamics through CytK as compared with α-hemolysin, we wanted to investigate whether enzyme-free translocation signals could provide the resolution necessary for extracting domain-level information. To investigate this, we designed PBI analytes P1-P4 (shown in Fig. 3a and Supplementary Table 1). Like MBP, each analyte has a C-terminal D10 tail. Each construct is comprised of two different ~70 amino acid (aa) sequence blocks from MBP repeating three times. These four PBIs were designed to be split into two isoform pairs, where P1 and P2 form one pair, and P3 and P4 form the other. Each PBI pair shares the same type and number of sequence block repeats but differ in how their repeat blocks are ordered (Fig. 3a). Both P1 and P2 contain the first 145 aa of MBP, which we divided into two sequence blocks, S1 (blue, 67 aa) and S2 (green, 78 aa). P1 has three repeats of S1 followed by S2 (S1-S2) to see if the blockade pattern produced by the first 145 aa of MBP could be detected three times within the P1 transport signal. P2 has three repeats of S2-S1 to see if 1. the signal pattern unique to S2 can be observed before S1 and if 2. the S2-S1 signal pattern can also be observed three times within the P2 transport signal. We used the same design rationale to make P3 and P4 PBIs, with the difference being that S2 was replaced S3 (yellow), which contains the last 69 a.a. of MBP (Fig. 3a).

DNA sequences for each PBI plasmid were designed and successfully expressed in BL21(DE3) *E. coli* as inclusion bodies, which were subsequently solubilized and purified via his-tag affinity chromatography using a Ni†^+^ column (see Methods and Supplementary Table 2). We confirmed that all four PBIs were successfully expressed and purified at their expected molecular weights with SDS-PAGE (see Supplementary Fig. 7). Although PBI pairs have the same molecular weight, the bands for P2 and P4 were located just below P1 and P3, respectively. However, this observation is consistent with previous reports showing that even single amino acid substitutions can affect a protein’s migration and position on SDS-PAGE.^63,64^ We further confirmed the expected molecular weight for each PBI with intact MS (Supplementary Table 4). We consistently observed a stable open-pore current for CytK at −300 mV and smooth translocation of MBP and all four PBIs, with instances of voltage reversals to unclog the pore (Fig. 3b). Reverse transport of P1-P4 through CytK was confirmed through voltage dependence experiments, where t_d_ decreased with voltage for each analyte (Supplementary Fig. 8–11). Increasing the voltage bias also resulted in a higher capture rate for all five analytes when normalized to concentration (Supplementary Table 3). The capture rates for MBP, P1, P3, and P4 at −300 mV were similar, within the range of 3.34 – 3.72 nM^−1^s^−1^. For P2, we observed a lower capture rate of 2.18–0.67 nM^−1^s^−1^ (Supplementary Table 3). Compared to the lower voltages (Supplementary Fig. 12–16), translocation events at −300 mV exhibit a higher SNR, which should improve protein fingerprinting accuracies.

### Domain-level current signatures of protein isoforms

The translocation signals of PBIs are influenced by multiple factors including the volume and charge of individual residues, their interactions with the pore wall, stretching, jamming and coiling of the peptide, and the various noise sources such as thermal fluctuations and parasitic capacitance. Despite the above, we find analytes of the same type to still exhibit transport signals of similar shape. To computationally investigate this, we first simplified our translocation current signals into blockade-level transitions, i.e., steps, using a Bayesian segmentation algorithm, and extracted the current mean, standard deviation, and duration as “features” for each step (Fig. 3c, see Methods). We note that with this approach, each event could have a different number of steps, which correlates well the dwell time (Supplementary Fig. 17). However, contributions from stochastic stalling are minimized in the segmentation process (Supplementary Fig. 18, see Methods). Depending on the analyte, we calculated an average translocation progression of 4–7 amino acids/step (Supplementary Table 5). We randomly selected 200 events from each protein type, and within each population calculated the pairwise Dynamic Time Warping (DTW)^65^ distance on step means and standard deviations, yielding distance matrices. Here, a few outlier events had a noticeably larger distance to the rest of the set. To assess the degree of similarity within each set, we deployed hierarchical clustering with “average” linkage criteria and sorted the original distance matrix based on branching height. The sorted matrices for the analyte types show one or two main clusters of events (appearing as low-distance squares), alongside the outlier events (Fig. 3ca). The main clusters used in the rest of this analysis are formed by setting a cutting threshold on the dendrogram at 80^th^ percentile of branching points and then selecting clusters that are larger than 10% of the starting population size (20 events) and combining them. For analytes that exhibit two large clusters, one of them is a variation of the event where the protein briefly dwells in the pore before fully exiting, showing a transient low-blockade level at the end of the event (Supplementary Fig. 19–23).

To identify a representative signal shape for each analyte, we used the barycenter averaging method with the Soft-DTW^66^ function as a distance metric (see Methods). Each barycenter represents the shape with the minimum soft-DTW distance to events in the pool, and its length matches the step count of the event with most steps in the cluster. The flowchart of the processing pipeline starting from raw events to barycenter computation is shown in Fig. 3d,e shows the barycenters of each analyte after clustering (black) with 7 random events warped onto them. Similar to sequence alignment problems, we note that when an event is warped onto its barycenter, we encounter three scenarios when aligning the steps: We define a “match” as a step in the event that is aligned to a step in the barycenter. An “insertion region” is where two or more steps of the event align to a single step in the barycenter. A “deletion region” is where a step in the event aligns to two or more steps in the barycenter. In the warped event representation, the deletion regions are stretched to span the length of their alignment target in the barycenter, and the insertion regions (of length x steps) are compressed to each span a length of 1/x.

**Extended Data Figure 4.**
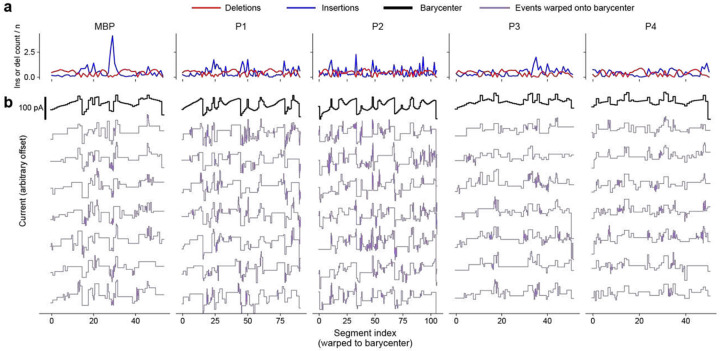
Realignment of segmented events onto the barycenters. **a,** Overall insertion and deletion score at each position, which are calculated the number of total insertions and total deletions observed at a particular index of the barycenter for each event’s alignment, divided by number of events. **b,** The barycenter of each protein type and seven events randomly chosen from the population used to generate the barycenter

It is common practice in genomic and transcriptomic analysis to report insertions and deletions by aligning to a reference. Similarly, we can treat the barycenter as a reference onto which events are aligned. Defining the set of consecutive steps of an event that align to a single point on the barycenter as an insertion site, and a single point of the event that aligns to a set of consecutive points on the barycenter as a deletion site, in Extended Data Fig. 4a we present an insertion/deletion map for all PBIs. Following aligning *n* events from each PBI’s pooled cluster of events, the total counts of insertions and deletions at all barycenter positions are divided by n, which may highlight areas that can be treated as landmark sites. Specifically, the barycenter positions where deletion rates are low or insertions are high can be deemed as common warping anchors.

In the P1 barycenter (Extended Data Fig. 4b), we see a triple-repeated shape which corresponds to its [S1,S2]_3_ composition. The first of these repeats closely matches the shape of the beginning of the MBP barycenter. Using this resemblance, we can identify the rough section of the MBP barycenter that corresponds to [S1,S2]. The P2 barycenter also shows a triple-repeating shape corresponding to [S2,S1]_3_. The beginning and end of this barycenter allow us to identify the approximate splitting point between S1 and S2 in the MBP barycenter. Across both P1 and P2, at regions where an S2 repeat ends and an S1 repeat begins, a new shape appears in the barycenter that does not exist in the MBP barycenter. This phenomenon is not surprising, since that particular sequence of amino acids representing the S2→S1 transition does not exist in MBP. This effect is even more clear when we look at P3 and P4, where the boundaries at [S1→S3] and [S3→S1] are all novel shapes, but the regions where the pore is likely sensing the central parts of S1 or S3 resemble the shape of those same segments in the MBP barycenter.

**Extended Data Figure 5.**
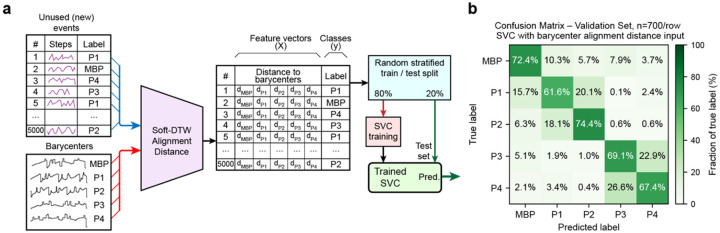
PBI classification using barycenter signal reference **a,** Machine learning pipeline describing usage of pre-derived barycenters for classification of new events. 4,000 events were aligned to each of the five barycenters, generating five distance values for each event. These distances were used as a feature vector for training and testing of a support vector machine classifier model. **b,** 5-way confusion matrix of the support vector machine classifier predicting the test set labels (correct-versus-all accuracy = 70.6%, versus 20% with random guessing).

If these barycenters truly capture the shape of each protein type’s blockade signal, one would anticipate them to be useful in predictive modeling and classification. To test this hypothesis, we developed a classification pipeline (Extended Data Fig. 5a). We sampled 5,000 events (1,000 per class) that were not used in the computation of the barycenters. Computing the Soft-DTW alignment distance of each event to every protein’s barycenter results in a vector of five distance values per event. This vector is the input “feature set” to a support vector machine classifier (SVC) tasked to identify the correct protein type. We used 80% of the events in training the model, and 20% in testing (validation). The confusion matrix of model calls on the validation set (Extended Data Fig. 5b) shows a correct-versus-all classification accuracy of 70.6%, significantly larger than a 20% accuracy expected from random guessing. This classification attempt simply demonstrates the validity of the barycenters as a “medoid” for alignment. While this exercise showed the predictive value of protein barycenters, any practical classification and protein identification requires significantly larger accuracies, which can be obtained through more information-rich feature sets and pipelines.

**Extended Data Figure 6.**
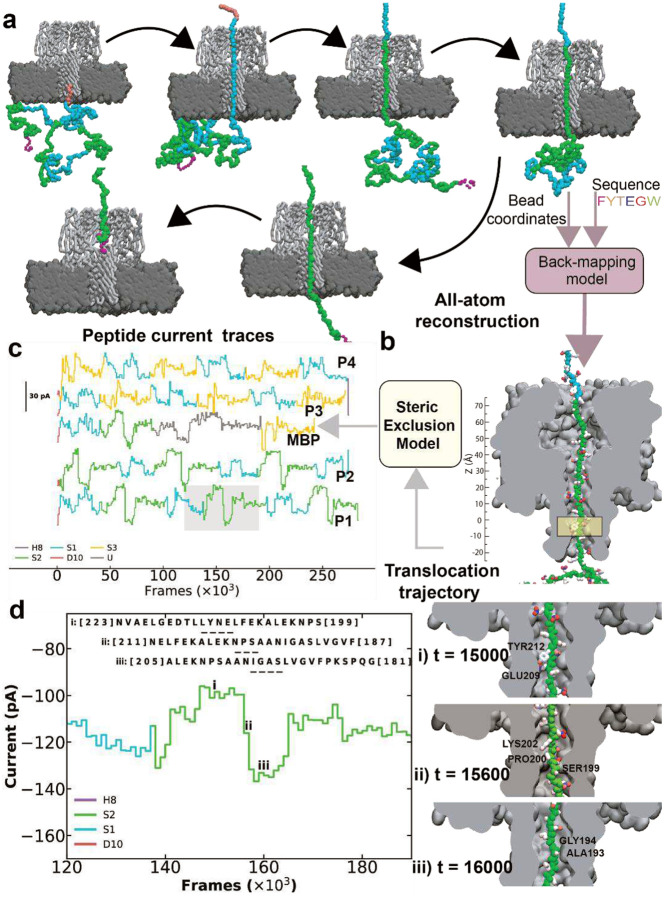
Multi-resolution workflow for full-length translocation current traces. **a,** Schematic of the trans-to-cis translocation at coarse-grained resolution. The P1 peptide is represented using spheres, with the residues belonging to S1 colored cyan, S2 colored green, the D10 tail at the C-terminus colored red, and the H8 tail at the N-terminus colored magenta. The steric grid of the lipid membrane is represented as a dark gray surface, whereas the coarse-grained model of the pore is drawn using silver bonds. **b,** Peptide’s coordinates and sequence are fed into back mapping algorithm to reconstruct the all-atom structure. The residue at the dipole constriction is highlighted. **c,** Block-averaged SEM current traces for five peptides, with the segments colored according to the residue at the dipole constriction. The current values have been averaged over 100 frames. **d,** Zoomed in view of the current trace in the gray box region of panel **c**. The sequence context for three current levels is shown above the plot, from C to N-terminus, consistent with the translocation direction. The numbers in the brackets specify the index of the first and last residue, respectively. The underlined residues are present in the dipole constriction, between z = −8 Å and z = 2 Å. The plots on the right show the conformations of the peptide at the indicated time steps, with the key residues near the dipole constriction labelled.

**Figure 4.**
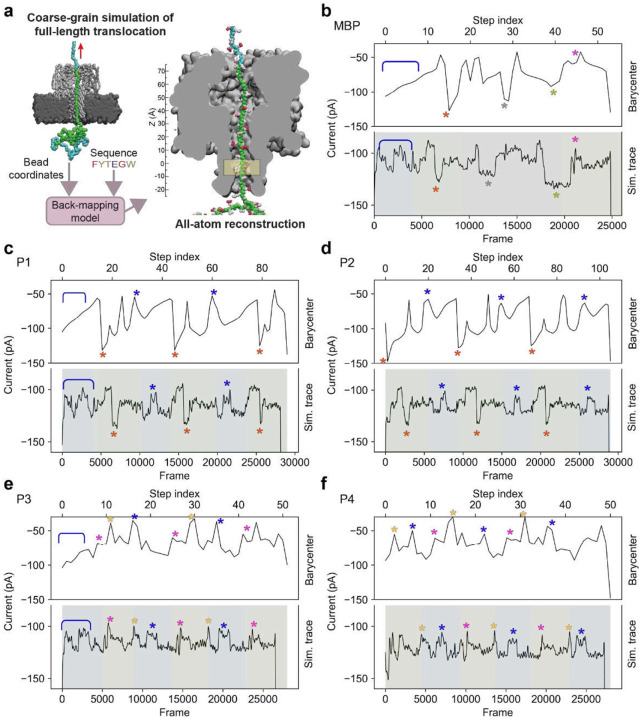
Multi-resolution simulations of PBI current signals versus experimental translocation barycenters. **a,** Instantaneous coarse-grained configuration (left) and the corresponding all-atom reconstruction (right) taken from a course-grained simulation of P1 transport through CytK (silver) The peptide backbone is represented using spheres, with the residues belonging to S1 colored cyan and S2 colored green. The sidechains are represented as cylindrical bonds, colored according to the partial charge of the atoms (blue for positive, red for negative and white for neutral). The highlighted region represents the dipole constriction. **b-f**, Experimental barycenter (top) and simulated trials of transport current signal (bottom) for MBP and all four PBIs with corresponding sequence blocks colored according to Fig. 3a. The simulated signals show some resemblance to the experimental barycenters. The discrepancy in both the mean and swing amplitudes between the simulated and experimental signals is likely due to differences between the model and actual structure of CytK and the excessive peptide stretching in simulations. Our interpreted similarities in simulated signals and barycenters are marked with a color-coded asterisk (*). The blue brackets reference the condition where segment S1 is at the beginning of the signal. The details of this portion are inconsistent and not visible in the barycenter, likely due to the influence of the strong electrophoretic force exerted on the D10 tail. The simulation process details are shown in Extended Data Fig. 6. A second replicate of the PBI simulated currents is shown in Supplementary Figure S24.

A computational framework capable of generating the expected signals of full-length proteins could lead to 1. agnostic fingerprinting of proteins and 2. accelerate the search for high-resolution nanopores. Taking advantage of the barycenters, we could assess the predictive power of simulated current signals generated with a coarse-grained + all-atom-remapped steric exclusion model (SEM) for each analyte (Fig. 4, Extended Data Fig. 6, Supplementary Figure S24, see Methods). Compared to the barycenters, the simulated signals exhibited lower fractional blockades and attenuated amplitude swings, however, both share similar trends in the signal’s pattern trajectory. Notably, the relatively large current enhancement observed in both P1 and P2 are also observed at the expected location within both their respective simulated trials (Fig. 4, Supplementary Figure S24). According to these simulations, this specific region within the signal is induced by the amino acids within the S2 block (Fig. 4, Extended Data Fig. 6, green). Furthermore, this particular pattern is not observed in either the barycenter or simulated signal of P3 and P4. These two PBIs, which do not contain the S2 block, exhibit an overall reduction in signal swings relative to P1 and P2, an observation consistent in both the barycenters and simulations.

### Deep-learning-based classification and inference on PBI translocation events

**Figure 5.**
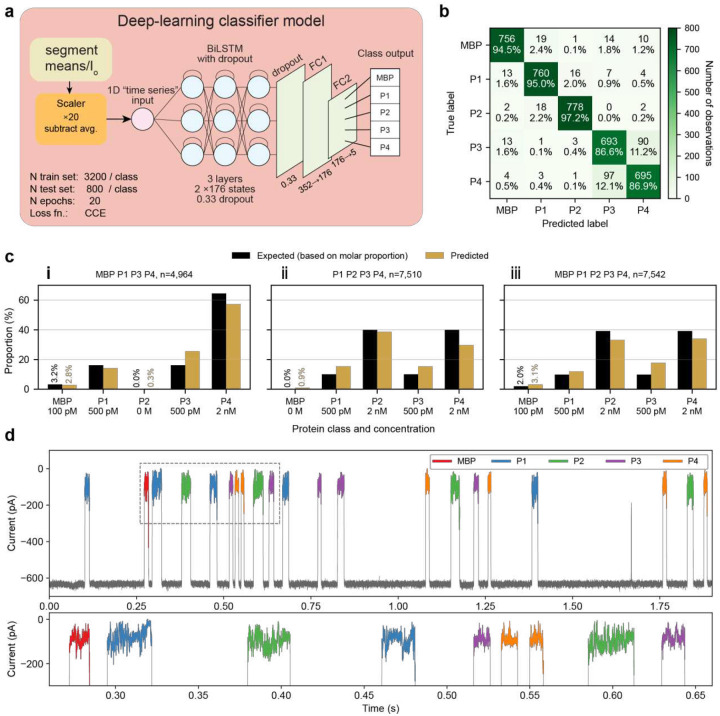
Deep-learning classification of protein isoforms. **a,** Architecture and parameters of the neural classifier model consisting of a BiLSTM section followed by a fully-connected (FC) section, leading to 5-way class outputs. The input to the model consists of the sequential set of step mean blockades steps for each event, scaled by a factor of 20 and normalized to a mean value of zero for each event independently. All events used here are recorded at −300 mV, have a duration between 2ms and 100ms, and contain more than 20 steps. **b,** Validation results of the trained model performing inference on the test set in a 5-way confusion matrix, showing a total accuracy of 92.05%. Percentage values are normalized by row (true class). **c,** Inference on three different mixture experiments, comparing the input molar proportion and predicted proportion. In c.i and c.ii, one analyte was left out, representing an expected value of zero. In c.i and c.iii, MBP was identified at a 100pM concentration within the mixture. **d,** Example ionic current trace of the 5-way mixture experiment displayed in c.iii, where the model predictions of events are used to color-code the trace.

As previously described, translocation events exhibit unique shapes which become more apparent when signals are averaged into a barycenter. This section describes a single-molecule classification approach using a recurrent neural network trained to discriminate among the five protein variants used in the study. Segmentation results in a different number of steps for each event, which necessitates the model to be compatible with variable input sizes. Recurrent neural networks are particularly attractive for variable-size, time-series-like inputs. The model architecture consists of a Bidirectional Long Short-Term Memory (BiLSTM)^67^ section with *L* layers, *H* hidden state size per layer and per direction, *D* dropout, followed by a classification head consisting of two fully connected layers (FC1 and FC2), mapping *2H* input size to *H*, and *H* to 5 classification output results, respectively (Fig. 5a). The model was trained and tested on MBP and P1-P4 translocation events recorded at −300mV bias, filtered for the individual events with a total duration within 2ms to 100ms, and minimum segment count of 21. 3,200 events from each class (16,000 events total) were used for training, and 800 events from each class (4,000 events total) were used for testing. The best hyper parameters for the model and learning schedule were found in a hyper-parameter search exercise using an automated script, which is the reason behind the odd values for the hyperparameters shown in Fig. 5a (details in Methods). Fig. 5b is the outcome of 5-way classification on the testing set (data excluded during training), showing a correct-versus-all accuracy of 92.05%. The primary source of inaccuracy across all training attempts was cross-confusion in discrimination between P3 and P4, which typically converged at correct call rates of 85% to 89%.

To validate the inference capacity of this model, we conducted experiments with mixtures of four or five proteins at different concentrations (Fig. 5c). The four-way experiments (Fig. 5ci–ii) are of particular interest for investigating miscall rates of the proteins that are not present in the mix, expecting zero calls. In both cases, miscalls of the absent protein occur at <1% proportion of all events. We note that the expected proportions displayed in Fig. 5c are based on molar ratio, disregarding the variations in capture rate. Based on isolated experiments, we would expect all proteins to have a similar capture rate except for P2, which was only half as efficient. However, the predictions in the mixture experiment are a closer match to the true molar ratio, and the common inconsistency observed is the apparent overcalling of P3 and undercalling of P4. We hypothesize that the proteins in the mixture, containing sequence blocks from MBP, can interact with each other in the denaturing solution, and these interactions may bias their capture by a small amount in a specific direction. Further investigation into co-capture or back-to-back capture of specific pairs of proteins could shed light on the phenomena causing these biases. In the example trace shown in Fig. 5d, model predictions of the identity of individual events from the five-way mixture recording are color-coded over the original (25 kHz filtered) trace. Note that the raw trace display is for visualization purposes only, and the model input only consists of normalized mean blockade values of steps resulting from segmentation (see Methods). These mixture experiments, designed for testing the limits of detection and classification, show that identification of a protein at a 100 pM concentration within a mixture containing 50 times the quantity of other proteins (5 nM) is feasible. However, detecting the total absence of a protein in a mixture is limited by our model’s accuracy, and at the current stage we observe a small fraction of false positives for the absent protein.

**Extended Data Figure 7.**
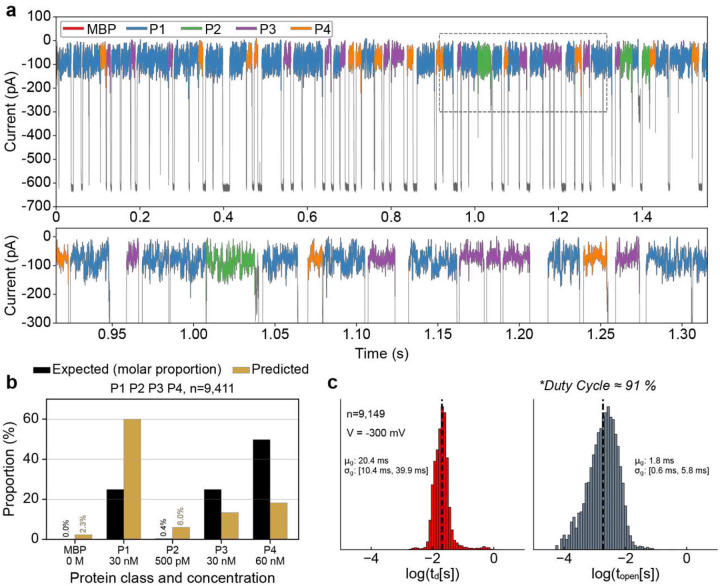
High duty cycle measurements under saturated concentrations retain protein discrimination. **a,** Representative continuous ionic current trace of a 4-way PBI mixture experiment with a total protein concentration of ~120 nM (V = −300 mV). The same model from Fig. 5 is used to predict PBI transport events under saturated conditions where an ultra-high event duty cycle is observed. Close-up portion of the signal shown below corresponds to the gray inset above. **b,** Inference on the 4-way PBI mixture experiment, comparing the input molar proportion and predicted proportion. **c,** Log-scaled dwell time distribution [log(t_d_)] of reverse transport events (left, red) and log-scaled interevent times [log(t_open_)] representing periods of open pore baseline (right, gray) obtained from high duty cycle mixture experiment shown in panel **a**. Dashed lines represent the geometric mean (μ_g_), with annotations indicating μ_g_ and the range of one geometric standard deviation (σ_g_).

The enhanced sensitivity enabled with reverse transport shows that above ~15 nM protein concentrations (Fig. 1e), measurements are saturated with a near-continuous event rate, potentially increasing the capacity to analyze target proteins within samples that contain high-concentration background proteins. Doing so, however, would require that the signal quality is not diminished. To investigate this possibility, we applied the model used in Fig. 5 onto a four-way PBI mixture experiment where the total protein concentration was 120.5 nM (Extended Data Fig. 7a,b). With MBP excluded from the mixture, the relative number of events predicted as MBP was the lowest (2.3%), indicating that the integrity of the transport signals is retained under saturated conditions. However, a substantial discrepancy was observed between the predicted and expected relative ratios for the four PBI analytes (Extended Data Fig. 7b). Similar to the hypothesis used to explain the increased capture efficiency observed with P2 in Fig. 5, the interactions between different proteins could alter their capture rates, which could be more pronounced at higher concentrations. Taking both the average translocation time (20.4 ms) and interevent time (1.8 ms), we find that our event duty cycle under saturated conditions is ~91% (Extended Data Fig. 7c), which, as far as we are aware, is the highest reported value. This result was further supported by the total current distribution, which shows a 93.5% relative occupancy for the blocked state (Supplementary Figure S25).

### Rapid quantification of full-length protein expression from crude cell lysates

**Figure 6.**
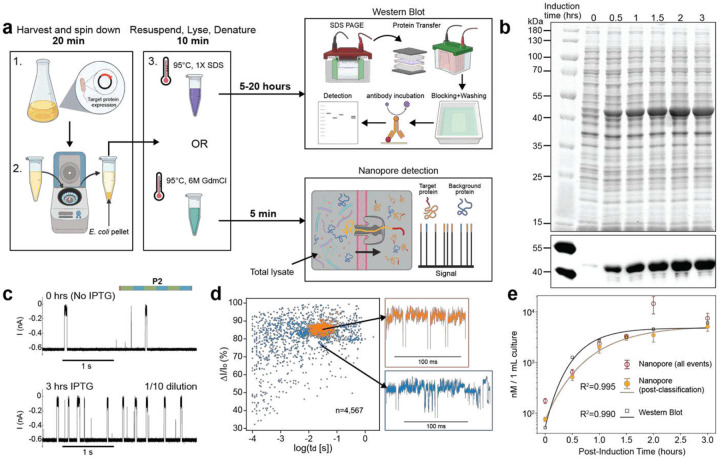
Detection and quantification of expressed protein in cell lysates. **a,** Sample preparation steps for analysis of protein expression in *E. coli* from crude cell lysate for Western Blot and nanopore. **b,** SDS-PAGE of P2 expression at various induction timepoints (top) and Western Blot (bottom). **c,** Nanopore measurements of P2 in reverse transport mode from *E. coli* lysates originating from cultures before IPTG induction (top) and 3 hours after IPTG induction (V = 300 mV, 3-hour sample diluted by 10x). **d,** Fractional blockade versus log-scaled dwell-time (t_d_) of translocation events from *E. coli* lysates. Events colored orange are translocation signals that align well to the barycenter reference for P2, while other events are colored blue. Example of translocation events that passed (orange, top) and failed (blue, bottom) to align to the P2 barycenter reference signal (right). **f,** Concentration of expressed P2 in 1 mL of *E. coli* culture versus IPTG induction time analyzed with Western Blot (black) and our nanopore approach (orange). Concentration values (Mean – SEM) determined using our method before (red) and after (orange) the removal of events that did not align well to the P2 barycenter signal reference. Solid lines show the Gompertz growth model fitting to both datasets (see Methods), with R^2^ values for each fit annotated.

High-throughput analysis of full-length proteins with nanopores offers a potentially faster way to detect and quantify the expression of synthetic proteins in cells. While current approaches such as SDS-PAGE and Western Blot involve multiple hands-on steps, they have the benefit of analyzing the product within the crude cell lysate, bypassing the need for a purification step. It was recently demonstrated that expressed proteins within a clarified lysate could be detected using a nanopore following cell lysis via sonication, cell debris removal by centrifugation, and overnight denaturation in GdmCl.^68^ Although promising, circumventing the preparation of a post-lysis supernatant to directly analyze protein expression within crude cell lysates would be a major improvement to nanopore-based approaches. This would require that both the pore and membrane stability are retained in the presence of complex cellular debris. To this end, we assessed whether the enhanced capture provided with a C-terminal D_10_ tag was sufficient to quantify protein expression directly from total cell lysate, and whether the complex mixture of matter in the lysate would disturb or interfere with our nanopore platform. We also benchmarked our method against Western Blot. We used both methods to determine expression levels of P2 in *E. coli* BL21 (DE3) cells before and at various times after induction of expression by isopropyl β-D-1-thiogalactopyranoside (IPTG) (see Methods). IPTG inactivates the LacI repressor and induces the expression of T7 RNA polymerase, which then transcribes the P2 gene located downstream from the T7 promoter. We grew P2-transformed DE3 cells at 37 °C for 3 hours and spun down 1 mL cultures before (0 hours) and after IPTG induction at 0.5, 1, 1.5, 2, and 3 hours (Fig. 6a). For SDS-PAGE and Western Blot sample preparation, cell pellets were resuspended in 1X SDS and boiled at 95 °C (Fig. 6a). Prior to nanopore measurements, pellets were resuspended in 6M GdmCl and boiled at 95 °C (Fig. 6a). Subsequent steps for analyzing the different P2 induction time points with Western Blot are illustrated in Fig. 6a, which can take between 5–20 hours depending on the blocking and antibody incubation times. For our method, we found that the addition of whole cell lysate into the *trans* chamber did not compromise of the stability of the membrane, even under high voltage conditions. This result demonstrates the possibility that the target protein can be detected immediately after the lysis and denaturation, offering a significantly faster workflow to confirming expression (Fig. 6a).

Prior to Western Blot, expression levels at different induction time points were analyzed with SDS-PAGE stained with SYPRO Ruby. Bands at the expected MW confirmed P2 expression after IPTG induction (Fig. 6b, top), however, it was not until we ran a Western Blot with anti-His tag antibody that we observed a band that leaky expression prior to IPTG induction (Fig. 6b, bottom. Next, we ran all P2 expression time points using our nanopore platform and observed translocation events, including the culture that contained leaky expression of P2 (Fig. 6c). Since background biomolecules in lysates were also sampled by the pore (Fig. 6d, blue), we added a classification step to computationally select and quantify only the target P2 protein. Each event was aligned to the barycenter reference signal for P2 using Soft-DTW. Events that had a Soft-DTW distance score >1,000 (see SI Figure S26) were not classified as a P2 protein molecule. This allowed for translocation events that exhibited a signature close to the expected P2 signal to be considered for subsequent analyses (Fig. 6d, orange). With this fingerprinting pipeline, we were able to extract the P2 mean capture rates in the lysate as a function of time which provided P2 concentration values. Remarkably, our results agree well with Western Blot quantification across the different induction time points (Fig. 6e). As shown in Fig. 6e, the model fits for P2 expression concentration versus IPTG induction time measured with our nanopore system closely matches the protein levels quantified by Western blot (see Methods), confirming that our method offers a reliable way to detect and quantify protein expression in a fraction of the time required to run SDS-PAGE and Western Blot.

## Discussion

Protein transport through nanopores was first demonstrated more than a decade ago,^41^ and since then, no alternative method capable of directly reading full-length proteins has emerged. With this unique mode of sensing, nanopore proteomic techniques can bypass peptide fragmentation steps needed for methods that rely on MS and Edman degradation. The most effective strategies for proteoform-aware analysis hinge on reading full-length, intact proteins and their constituent PTMs.^21,69,70^ On the other hand, nanopore proteomics has yet to address the major challenge that MS methods are actively working on, which is the need for high-throughput measurements capable of detecting billions of proteins in a sample, while maintaining the sensitivity to overcome the wide dynamic range in protein abundance, which can span 10–12 orders of magnitude.^25,71–73^

Among the various remaining challenges, increasing the overall dynamic range by orders of magnitude^74^ and achieving true sequence-signal relationship in proteins are far in the distance. While enzyme-driven approaches can detect nuanced changes in amino acid compositions such as detection of differences in signals for single-amino acid substitutions and PTMs in known peptide and protein sequences, they come at the cost of low throughput. This is because they are limited by the motor’s slow stepping rate, which can take >10 seconds to unfold and translocate a single protein through a nanopore.

In this study, we discovered that reverse transport, where protein capture proceeds from the β-barrel of CytK, enhances the sensitivity of our approach by 2.5 orders of magnitude as compared to forward transport (Fig. 1e). This increased efficiency in reverse protein capture allowed the first demonstration of unfolded protein recapture in a nanopore (Fig. 1g–i). Initially, this large difference in capture efficiency was unexpected considering that the open-pore conductance of CytK under negative biases was only 2.5% higher compared to positive biases (Fig. 1b). Employing MD simulations revealed distinct EOF profiles within the lumen of CytK at positive and negative biases. Specifically, the increased capture rates at negative biases could be explained by the higher Gdm^+^ density near the entrance of the β-barrel of CytK. This localized increase in Gdm^+^ concentration is induced by the negatively charged Glu141 ring, where Gdm^+^ can readily bind under negative biases because of the enhanced dipole moment of Lys130 and Glu141 residues (Fig. 2c,d). We additionally verified that the enhanced capture efficiency observed with reverse transport results from denatured proteins adsorbing to the lipid membrane. Through this mechanism, surface diffusion of proteins along the membrane pre-concentrates them near the pore, which facilitates capture. Compared to the vestibule, the pore opening from the β-barrel side of CytK is more accessible for membrane-bound proteins. The combined effects of membrane affinity and the increased electric field at the mouth of the β-barrel side yield a ~2.5 orders of magnitude enhancement in relative capture rate for reverse vs. forward transport.

Despite the much lower charge density of proteins as compared with nucleic acids, reverse transport of proteins through the β-barrel of CytK proceeds with high rates that are on par with estimated capture rates from nanopore sequencing. For a typical input of 100 ng mRNA in RNA004 MinION flowcell the RNA concentration is ~0.1–1 nM, and the number of reads is on the order of ~10^6^ per day,^75^ which translates to 10^3^ reads per pore per day. In turn, our enzyme-free protein fingerprinting approach achieves a measurement rate of >10^4^ per day at 0.1 nM concentration, which favorably compares with the enzyme-driven DNA/RNA sequencing. Furthermore, the sensitivity of our approach allows rapid (~1 min) protein detection at pM concentrations (Fig. 1e and 5c) with a minimal chemical tag (D_10_), which offers the potential to detect low-abundance proteins within the broad dynamic range inherent to complex proteomes while retaining the full-length context of proteins.

Along with a vast improvement to protein readout rates, we show here that signal post-processing can mitigate the effects of dwell time stochasticity associated with our quasi-regulated enzyme-free transport. The decreased transport speeds with CytK, as compared with α-hemolysin, permits measurements at higher voltages (Fig. 1f, Fig. 3b), thereby enhancing the signal-to-noise ratio of our measurements. Our ability to transport proteins through CytK at high voltages by using Gdm^+^ ions to induce an EOF, as opposed to relying on charged residue substitutions within the pore lumen, underscores the importance of the mechanism used to generate an EOF through the pore. In particular, recent work demonstrated that protein transport through a CytK-4D mutant in presence of urea was limited to 120 mV, as the majority of events were long-lived blockades at higher voltages, indicative of protein clogging.^52,54^ Using this favorable interplay between slowed transport dynamics and high-voltage measurements, we developed a pipeline where segmented signal levels can be clustered prior to implementing alignment schemes that can extract and map the regional fingerprint of proteins (Fig. 3 and Fig. 5). This allowed for reproducible domain-level signatures to be captured within the signal of PBI analytes designed with blocks of amino acids taken from MBP (Fig. 3). Complementing machine learning analysis of experimental traces, our proof-of-principle multi-resolution simulations show that accurate, purely computational prediction of ionic current signatures of full-length protein translocation is well within the reach of current computational technology. Moreover, we were able to train a recurrent neural network and recover the relative ratios of PBI analytes within a mixture. This type of predicative power is incompatible with MS methods since the fragmentation of all five proteins would make it impossible to map peptides back onto the sequence blocks corresponding to the original analyte since these blocks are shared across all five protein isoforms. Finally, we showed the stability of our system after adding crude lysate to the *trans* chamber and demonstrated detection and quantification of protein expression at different induction time points. We were able to determine the protein concentration from miniscule amounts of *E. coli* culture and found good agreement with Western Blots. The high sensitivity of our method also allowed for the quantification of leaky protein expression, which remained unobservable with SDS-PAGE and required further analysis with Western blot.

Several challenges remain for this technology to advance the field of proteomics. First, methods for substrate-agnostic conjugation of charged tails to the termini of full-length native proteins at high yields are still immature. In this work, we relied upon proteins with co-expressed purification and capture tags to streamline our interrogation of signal quality. However, our findings and methods can conceivably extend to native proteins; we previously demonstrated chemical tagging and translocation of full-length proteins using DNA oligos^50^ and have been in pursuit of high-yield, specific chemical tagging approaches.^76^ Second, the resilience of the membrane must be improved to withstand higher Gdm^+^ concentrations (up to 6M^77^) that may be needed to unfold ultrastable regions within the proteome. As a workaround, we previously denatured tightly folded proteins in heated 6M GdmCl solution and diluted the pre-denatured proteins in a 2M GdmCl nanopore chamber.^50,58,78^ Third, while our results demonstrate that domain-level signatures of a protein can be resolved without an enzyme, achieving accurate amino acid identification and PTM mapping will require nanopores with narrower constrictions to reduce the spatial size of the k-mer window. Despite these limitations, we resolved and quantified here block isoforms at high sensitivity and corroborated measurements with simulations. This demonstration of a proof-of-concept proteoform-level resolution is a further step towards overcoming some of the existing challenges and enabling the analysis of more complex aspects of the proteome, with potential implications for life science and biopharma research practices and ultimately, in the clinic.

## Methods

### Single-channel experimental setup

Single-channel recording experiments were done with a PTFE flowcell, which consists of two chambers separated by a 25 μm-thick PTFE sheet with a 70 μm diameter aperture in which the lipid bilayer is formed. Before bilayer formation, the aperture was pretreated by applying a small droplet of 2% n-hexadecane in n-pentane. Next, each chamber was filled with 500 μL of the experimental buffer (1M KCl, 2M GdmCl, 10mM Tris, pH 7.5), which has a conductivity of 240 mS/cm at room temperature. Then, two 0.5 μL droplets of 1,2-diphytanoyl-sn-glycero-3-phosphocholine (DPhPC) lipid (5 mg/mL in n-pentane) were added onto the surface of the buffer in each chamber and gently mixed with a 1 ml micropipette to spread the lipid without introducing bubbles. After lipid application, Ag/AgCl electrodes were inserted into both chambers and bilayer formation was achieved using Montal–Müller painting/aspiration, where the buffer was slowly withdrawn and then aspirated back into the chambers until a stable bilayer spanned the aperture.^79^ After bilayer formation, a voltage was applied to confirm that there was no leakage current. Next, ~0.2 μL of CytK stock (15 μg/mL) was added to the *cis* chamber to achieve single-channel insertion. After CytK insertion, the offset voltage was corrected, followed by the addition of the protein analyte into the *trans* chamber for reverse translocation measurements and/or into the *cis* chamber for forward translocation measurements. For the recapture experiments, the protein analyte was added to the *cis* chamber only after successful membrane formation and single CytK insertion. The recapture experiment proceeded as follows: a positive voltage was applied until a protein molecule was captured and translocated from the *cis* to the *trans* chamber. A 2 ms delay was applied after translocation to ensure that the protein fully exited the pore stem and entered the *trans* chamber. Next, the voltage polarity was inverted to a negative bias, allowing the same protein molecule in the *trans* chamber to be recaptured and translocated back into the *cis* chamber. The negative voltage was applied for 500 ms to facilitate the recapture attempt. All experiments were recorded with an Axopatch amplifier set to a gain of 10, with signals digitized at 16-bit resolution and a 250 kHz sampling rate using a National Instruments data acquisition card. Custom LabVIEW software was used to record and store the raw current and voltage data together with the acquisition parameters.

### Plasmid design, expression, and purification of PBI protein analytes

To generate PBI analytes, plasmids encoding PBI proteins were designed with DNA sequences that ensured repetitive protein segments had limited DNA sequence similarities while maintaining *E. coli* codon frequency usage. Plasmids were ordered through Twist Bioscience. Prior to ordering plasmid designs, sequences were uploaded to Twist’s online platform and designs flagged for high repeat dense areas were further optimized using the Twist Codon Optimization tool. All PBI analytes were designed with an N-terminal 8X-histidine tag for IMAC purification and a C-terminal 10X-aspartic tag for efficient capture and unidirectional translocation.

Prior to PBI expression, PBI plasmids were amplified. Plasmids were transformed into NEB 5-alpha Competent *E. Coli* (C2987H) following the manufacturer’s instructions and selecting on LB agar plated containing 100 μg/mL ampicillin at 37 °C. Single colonies were inoculated into separate flasks of 30 mL of LB medium containing 100 μg/mL ampicillin and grown at 37 °C with shaking at 220 rpm for 16 hours (Eppendorf Innova 42 and 42R). Plasmids were isolated from cells using PureLink quick Plasmid Miniprep Kit and sequence-verified by whole-plasmid sequencing (Plasmidsaurus).

Each PBI plasmid was transformed into NEB BL21 (DE3) cells following the 5-Minute heat shock transformation protocol. Cells were grown overnight at 37 °C on agar plates containing 100 μg/mL ampicillin. To make primary cultures, single colonies were inoculated into separate flasks with 30 mL of LB medium containing 100 μg/mL ampicillin and grown at 37 °C while shaking at 220 rpm for 16 hours. To make the secondary culture, transformed cells from the primary culture were transferred into 500 mL of LB medium containing 100 μg/mL ampicillin in a 2.8 L baffled Fernbach flask, starting at an optical density at 600 nm (OD_600_) of 0.01, and incubated at 37 °C with shaking at 220 rpm. When the culture reached an OD_600_ of 0.6, protein expression was induced with 1 mM IPTG. PBI proteins were expressed as inclusion bodies (IBs) by continuing incubation at 37 °C with shaking at 220 rpm for 3 hours. After induction, cultures were placed on ice for 30 minutes, then centrifuged at 3,000 × g for 20 minutes. Cell pellets were decanted and stored at −80 °C.

Each cell pellet was resuspended in 25 mL of ice-cold lysis buffer (Table 1) and transferred to a steel beaker placed in an ice water bath. Cells were lysed by sonication using a Fisher Scientific 550 sonicator at 70% amplitude with a 2-second on / 4-second off pulse cycle for a total of 3 minutes. The resulting lysate was transferred to a 50 mL Falcon tube and centrifuged at 12,000 × g for 10 minutes at 4 °C. The supernatant was discarded, and the pellet was resuspended in 25 mL of wash buffer 1 (Table 1), transferred back to the steel beaker, and sonicated under the same conditions for 1 minute. After centrifugation at 12,000 × g for 10 minutes, the supernatant was again discarded. The pellet was then resuspended in 25 mL of wash buffer 2 (Table 1), followed by a final round of sonication and centrifugation under the same conditions. The resulting pellet contained isolated IBs, which was then stored at −80 °C for future purification. Note that the final IB pellet should appear white.

For IMAC purification, IB pellets were first solubilized by resuspending in 10 mL of IMAC wash buffer (see Table 2) and mixed via tube revolver set to 4 °C for 1 hour to denature the IB pellet in 6M GdmCl. A Cytiva 5 mL HisTrap HP prepacked Ni^2+^ column connected to an ÄKTA pure^™^ FPLC system was equilibrated with degassed 1. Milli-Q water, 2. IMAC elution buffer, and 3. IMAC wash buffer, in that order. Each step was 5 column volumes (CV) at a 5 mL/min flow rate. Next, the solubilized analyte filtered with a 0.2 μm PVDF syringe filter and loaded into the HisTrap column at 1 mL/min. Non-specific E. coli proteins were removed from the column with 10 CV of IMAC wash buffer (5 mL/min). Target analyte was then eluted with a 5 CV linear gradient from 0% to 50 % IMAC elution buffer (5 mL/min), and 2 mL fractions were collected. Fractions with the highest A_280_ were pooled together, concentrated using a Pierce 10 kDa MWCO PES centrifugal concentrator, and buffer-exchanged into 6 M GdmCl, 10 mM Tris pH 7.5. The PBI analyte concentration was determined with an Eppendorf 6135 BioSpectrometer, after which samples were aliquoted and stored at −80 °C for downstream nanopore experiments. To prevent any cross-contamination, each PBI analyte was purified with a separate, dedicated HisTrap column. Note that all data collected in this study for each PBI came from a single round of expression and purification.

### Expression and purification of CytK

The plasmid encoding WT CytK was kindly provided by Dr. Giovanni Maglia at the University of Groningen. Following the protocol as described in Sauciuc *et al*.^52^ CytK was expressed in *E. coli* BL21(DE3) cells grown in LB medium supplemented with 100 μg mL^−1^ ampicillin for 21 hours at 25 °C while shaking at 180 rpm. Next, cells were lysed and CytK was purified via Ni^2+^–NTA affinity chromatography.

### Dwell time distribution KDE fitting

The probability density function (PDF) of dwell times for each analyte was estimated using the gaussian_kde function from scipy. The KDE represents the PDF as a sum of Gaussian kernels centered at the observed data points and is defined as:

$$f_{h}\left(x\right)=\frac{1}{n}\sum_{i=1}^{n}\frac{1}{\sqrt{2\pi }h}e^{\left(-\frac{{\left(x-Xi\right)}^{2}}{2h^{2}}\right)}$$

where *n* is the number of samples, *h* is the bandwidth (smoothing parameter), *XI* are the observed data points, and *x* are the positions at which the density is evaluated. The evaluated *x* positions were generated through the linspace function in numpy, with 1000 equal increments between the minimum and maximum *Xi* values in the dataset. The bandwidth parameter, *h*, was calculated using Scott’s rule, which scales the kernel width according to the variance and sample size of the distribution, which is defined as:

$$h=\sigma n^{-\frac{1}{d+4}}$$

where *σ* is the standard deviation of the distribution, *n* is the number of samples, and *d* is the number of dimensions, which is 1. The table below shows the bandwidth values used in the KDE fit for log-scaled dwell time for each analyte across different voltages

**Table:** 

Analyte	Transport orientation	150 mV	175 mV	200 mV	225 mV	250 mV	275 mV	300 mV
**MBP**	Forward	0.220	0.134	0.103	0.106	0.064	0.076	0.067
**MBP**	Reverse	0.065	0.060	0.058	0.074	0.10	0.083	0.081
**P1**	Reverse	0.161	-	0.095	-	0.123	-	0.115
**P2**	Reverse	0.108	-	0.084	-	0.077	-	0.088
**P3**	Reverse	0.074	-	0.076	-	0.057	-	0.052
**P4**	Reverse	0.077	-	0.056	-	0.050	-	0.059

### Capture rate versus concentration experiments and analysis

For every experiment with a specific concentration of MBP or PBI analyte, the required volume of protein stock was added to both *cis* and *trans* chambers. For capture rate analysis, recordings were segmented into continuous voltage steps, where each step starts and ends upon the application of a voltage change or reversal. The events and inter-event times within each voltage step were extracted. The inter-event time was defined as the time between the start of each event and the start of the preceding event. Next, the capture rate for each voltage step was calculated as the inverse of the mean inter-event time. Events were included in the analysis if they had a fractional blockade level greater than 70%, and a t_d_ between 3 ms and 200 ms. The final capture rate calculated for each concentration was calculated as the weighted mean – standard dev. across all the continuous voltage steps for each analyte, with the weights proportional to the number of events parsed for each voltage step.

### MD Simulations

#### General MD methods.

All-atom MD simulations were performed using the GPU-resident NAMD 3.0 software^80^ and the CHARMM36 force field^81^ enhanced with the CUFIX corrections^82^ for accurate description of ion interactions. Water molecules were described using the TIP3P model.^83^ A 2 fs integration timestep was employed, with all covalent bonds involving hydrogen atoms constrained using the SHAKE algorithm,^84^ and water geometry constrained using the SETTLE algorithm.^85^ Van der Waals and short-range electrostatic interactions were calculated using a cutoff of 12 Å with a switching function starting at 10 Å. Long-range electrostatics were evaluated via particle-mesh Ewald (PME) summation.^86^ Temperature was maintained at 295 K using Langevin dynamics^87^ with a 1 ps^−1^ damping coefficient applied to all non-hydrogen atoms of lipid molecules. A semi-isotropic pressure coupling was applied during the equilibration via the NosØ–Hoover Langevin piston method^88^ to maintain 1 atm pressure and the constant ratio of the unit cell dimensions within the plane of the lipid membrane (the x-y plane).

#### System preparation and equilibration.

The heptameric CytK model was based on PDB ID 8RJ8^54^ and optimized using Rosetta.^89^ The optimized all-atom structure of CytK nanopore was embedded into a pre-equilibrated 14.5 nm × 14.5 nm patch of DPhPC bilayer membrane. Lipid molecules overlapping with the nanopore were removed. The resulting system was solvated following which potassium and chloride ions were added to neutralize the system and bring the ion concentration to 1 M. The system underwent energy minimization for 1,000 steps using the conjugate gradient method, followed by a 100 ps equilibration with positional restraints applied to all non-hydrogen atoms of the protein and lipid molecules. Subsequently, a 100 ns equilibration under constant number of particles, pressure and temperature (NPT) conditions was performed, with harmonic restraints (0.1 kcal mol^−1^ Å^−2^) applied to the Cα atoms of the protein. Taking the last frame of the equilibrated system, an appropriate number of Gdm^+^ and Cl^−^ ions were randomly introduced in the electrolyte solution to achieve 2M GdmCl / 1 M KCl electrolyte mixture. The resulting system was 14.1 × 14.1 nm × 14 nm in size and contained 291,943 atoms. The system underwent energy minimization and equilibration as described above, followed by a 100 ns NPT equilibration under the same Cα restraints.

#### Open pore current simulations.

A transmembrane voltage of desired magnitude and polarity was generated by applying a constant external electric field normal to the lipid membrane.^90^ The electric field magnitude was computed as *E*_*z*_=-*V/L*_*z*_, where *V* is the transmembrane voltage and *L*_*z*_ is the length of the simulation unit cell along the direction of the applied field. Production simulations were carried out for 300 ns under constant number of particles, volume and temperature (NVT) conditions with harmonic restraints (0.1 kcal mol^−1^ Å^−2^) applied to the protein’s Cα atoms.

#### Peptide translocation simulations.

To model peptide transport, the pre-equilibrated open pore system was extended by adding electrolyte of matching ion composition large enough to accommodate the extended peptide chain and avoid interactions with the nanopore over the periodic images of the unit cell. The resulting system (~0.35 M atoms) was minimized and equilibrated for 1.5 ns while restraining all non-hydrogen atoms of the lipid membrane and the nanopore. Two nanopore-peptide systems were prepared by placing a stretched 20-residue peptide on either side of the nanopore stem. The peptide sequence matched the sequence of the first 20 amino acids of the experimental MBP construct, inclusive of the D_10_ tag. In both systems, the D_10_ terminus was oriented toward the pore opening. Water and ions overlapping with the peptide were removed while preserving the overall ion concentration. Following the above minimization and stepwise equilibration protocol, each system was equilibrated for 50 ns under NPT conditions applying harmonic restraints on all Cα atoms of the peptide and the nanopore. Thereafter, 24 independent replica simulations were run in parallel for each configuration under a constant transmembrane potential of 1.2 V. The systems with the peptide placed at the cis side were simulated under +1.2 V (“cis-to-trans” translocation direction), while those with the peptide on the trans side were simulated under −1.2 V bias (“trans-to-cis” translocation direction). These simulations were performed under NVT conditions with the Cα atoms of the nanopore restrained as described above. During these simulations, the Cα atoms of peptide residues 1, 5, 10, 15, and 20 were harmonically restrained (5 kcal mol^−1^ Å^−2^) to remain at the pore central axis (the z-axis), while the peptides were allowed to move along the z-axis.

#### *In silico* measurement of the effective force.

To characterize the force experienced by the peptide during the translocation, seven configurations were generated for each translocation direction (forward and reverse). In each configuration, the leading residue’s Cα atom was positioned at 10 Å intervals along the pore axis, covering a translocation path from −20 Å to +40 Å. The following two restraint schemes were employed to minimize nanopore-peptide interaction and expedite the simulations. In both schemes, the Cα atom of the leading residue was restrained in all three spatial dimensions. In Scheme I, the Cα atoms of residues 5, 10, 15, and 20 were restrained only in the x and y directions, allowing them to adjust their z coordinates. In Scheme II, the Cα atoms of residues 1, 5, 10, 15, and 20 were restrained along all the three axes. The spring constats of the restraints was 5 kcal mol^−1^Å^−2^ for each restrain direction. The instantaneous force experienced by the peptide was taken as the sum of z-components of the restrain forces applied to all z-restrained atoms multiplied by −1. Three independent replicas were run for each restraining protocol and the peptide placement. The average force at each restraint position was obtained by computing the mean slope of time-integrated force plots across the three replicas. The associated error was estimated via error propagation, using the standard error calculated over 30 ns trajectory segments within each replica.

#### Trajectory analysis.

Visualization and trajectory analysis were performed using VMD.^91^ Instantaneous ionic currents and water fluxes were calculated as previously described.^90^ The ionic current and water fluxes were calculated within a cylinder of 20 Å radius coaxial with the pore axis, in the volume occupied by the CytK stem, i.e., from - 20 Å≤ z ≤ +40 Å. Average values and associated errors were obtained from the slopes of time-integrated plots, with standard errors computed across 30 ns trajectory fragments. Error propagation methods were used to compute uncertainties over the replicas. Electrostatic potentials were calculated as the time average of instantaneous potential maps using the PMEpot plugin^90^ in VMD.

### Coarse-grained simulation of peptide transport.

All coarse-grained (CG) simulations were performed using Atomic Resolution Brownian Dynamics (ARBD).^92^Each amino acid was represented using a one-bead-per-residue Calvados3^93^ model. All production simulations were carried out using a 10 fs time step whereas the initial relaxation of each system’s coordinates was done using a time step of 0.1 fs. Prior to the simulation of peptide translocation, the beads representing either the MBP-D10, or synthetic P1, P2, P3 or P4 peptides were initially placed along a vertical line 3.8 Å from each other. The C-terminus D-10 tail was placed close to the trans side opening of the nanopore. The center of mass of the D-10 tail was then restrained using a harmonic potential (k‹spring = 10 kcal mol-1 Å-2) to this point and the system was equilibrated for 100 ns after initial 1000-step relaxation. The nanopore translocation of peptides was realized by using a harmonic spring bond (k‹spring = 10 kcal mol-1 Å-2) that applied between the center of mass of the C-terminus D-10 tail a fixed dummy particle located approximately 1400 Å away from the top of the pore, depending on the peptide length. The spring rest length was then shortened at a rate of 5 Å /ns, producing peptide translocation at~ 1.5 amino acids/ns. The translocation simulations were run for 290 ns. The instantaneous coordinates were recorded every 10 ps.

#### Course-grained model of CytK / membrane system.

The one-bead-per-residue model for the pore was built using the last frame of the 250 ns MD simulation of the all-atom structure carried out at −250mV. Each bead was placed at each residue’s center of mass. All beads representing the nanopore were restrained to their initial coordinates using a harmonic potential (10 kcal mol^−1^ Å^−2^).

Test simulation showed that steric interactions between the pore and the peptide were not sufficiently strong in the Calvados3 model, allowing the beads to come too close to one another, which caused clashes during conversion to all-atom resolution. To remedy the problem, we introduced a grid-based potential to prescribe proper steric confinement on the translocating peptide. The potential was obtained using the volmap plugin VMD^91^ that calculated the distance map at each point in space from the edge of the nearest nanopore atom. The distance map was then used to build a steric map of the pore for each of the twenty amino acids as *U* = *k*(*r*− *r*_0_)^2^, where k was 2 kcal mol^−1^ Å^−2^ and *r*_0_ was the radius of the amino acid bead in the Calvados3 model. The resulting values of the potential varied between 0 and 25 kcal/mol and the potential applied only to the bead of the translocating peptide. Similarly, a lipid membrane was modeled using a steric grid potential defined on a 300 Å × 300 Å × 28 Å rectangular slab containing a hole at its center to accommodate the nanopore. The hole was manually carved such that the lipid membrane potential would not extend into the nanopore lumen. The lipid membrane potential varied from 0 to 20 kcal/mol and applied only to the beads representing the translocating peptide.

#### All-atom back mapping and current calculations.

Finally, we use the cg2all^94^ python package to obtain all-atom representations of our CG trajectories. The inference was performed using the weights for the center of mass-based reconstruction. Each frame of the all-atom trajectory was energy-minimized using NAMD 3.0 (cite Phillips, J. C. et al. Scalable molecular dynamics on CPU and GPU architectures with NAMD. J. Chem. Phys. 153, 044130 (2020).) in vacuum for 500 steps, removing steric clashes.

The resulting minimized all-atom trajectory was then used to calculate the ionic currents by means of the steric-exclusion model (SEM)^95^. The calculations used 1 Å resolution grid and the compute domain dimensions of 45 Å × 45 Å × 162 Å, to minimize the effects of access.^96^ The bulk conductivity for the SEM calculations was set to 20.0840 S/m, a value obtained from all-atom MD simulations of a 1M KCl / 2.5M GdmCl solution.

### Segmentation of translocation events

A parametric Bayesian segmentation algorithm implemented by Schreiber and Karplus^97^ and integrated into our data analysis package ionique (https://github.com/wanunulab/nanopore-pbi-fingerprinting) was used to segment signals. Briefly, the algorithm assesses the likelihood of a region of signal belonging to a single gaussian distribution or to two, and best splitting points are derived recursively. The Jupyter notebooks provided on GitHub illustrate this approach and the parameters used in detail. The chosen parameters of this algorithm over-segment the signals at first. In the subsequent cleanup step, through a recursive process, adjacent segments with the lowest difference in means are merged into one segment iteratively until the smallest difference in the resulting sequence is above a certain value (5 pA). For signals represented in dynamic-time-warping analyses in this work, the termination of iterations occurred when the number of steps dropped to the average starting number of steps in the events of that protein type. For signals used in neural-network classification analyses, due to the requirement for single-molecule decision making with disregard for the population, merging iterations terminated once the lowest difference in average blockade of consecutive steps in an event reached a value above 1%.

### Dynamic Time Warping, Barycenter Averaging, Alignment, and classification

To extract a barycenter representing the average shape of signals of a given protein type, the following steps were taken:

#### All-versus-all distance matrix and clustering.

200 events from each protein type were randomly sampled. Using sequences of segment means, an all-versus-all distance matrix was computed wherein each point in the matrix represented a the DTW distance of a pair of events in the set. Each DTW computation was constrained by a Sakoe-Chiba envelope with a radius of 20 steps. This 200 by 200 distance matrix was then used in agglomerative hierarchical clustering with an unweighted average linkage method (Unweighted Pair Group Method with Arithmetic mean, UPGMA). The resulting tree (dendrogram) was cut at the 80^th^ bottom-up percentile of branching points and all events within cut branches were grouped together to form clusters. Small clusters with a population less than 10% of the starting sample size (200) were dropped as outliers. Visual inspection revealed that these outliers, showing a large DTW distance to most other events, are typically instances of proteins getting stuck in the pore for a prolonged duration, effectively vibrating in place. The remaining branches were then merged into one large cluster for each protein class. The events in this large cluster were used in barycenter computation and alignment (insertion/deletion) analysis.

#### Barycenter computation.

The barycenters of each protein type were computed using the Soft-DTW barycenter averaging method, which uses the Soft-DTW distance metric to calculate through iterative minimization a time series with the lowest distance to all input traces.^66^ As parameters, a maximum of 5,000 iterations, a tolerance of 1E-8, and a smoothing parameter γ equal to 5 times the median length of event steps gave reliable results that were neither too smooth nor contained one-off shapes from individual events. This was confirmed visually through trial and error. Since this algorithm outputs a signal as long as the longest input sequence, it often produces flat regions in the barycenter. We shortened this signal by iteratively merging (averaging) pairs of consecutive points in the barycenter with the lowest difference until the length of the barycenter reached the median count of event segments. The resulting barycenter of each protein sample is displayed in Figs. 4 and 5.

#### Event alignment to barycenters.

For alignment, the barycenters were treated as references, and the pairwise Soft-DTW distance and alignment map between each event and barycenter were calculated. The alignment map, consisting of a sequence of pairs of indices (event segment mean position, barycenter position), was used to stretch or compress the event segment means for visualization, while leaving the barycenter the same. The compression of event steps occurs when multiple steps align to one point on the barycenter, also referred to in this work as an “insertion”. We displayed this compression by assigning fractional offsets to the x position of aligned points; for example, 3 segments of the event aligned to position 10 on the barycenter would span <9.5 to 9.83, 9.83 to 10.17 ,10.17 to 10.5>. stretched positions would simply span multiple points; For example, if a segment of an event is aligned to 4 positions, 15 through 18, it is displayed as a step spanning <14.5 to 18.5>.

### Classification of protein identities with a BiLSTM Neural Network Model

#### Preprocessing.

The step means of events were divided by open pore current to produce baseline-normalized levels. We found that when the events were scaled such that the combined population of the steps of all events had a mean of 0 and standard deviation of 1, experimental variations such as small leakages in the membrane would have a drastic effect on prediction outcomes and errors. To address this, we shifted the baseline-normalized mean of steps of each event to 0 and scaled the result by a factor of 20. To remove the extreme outliers, events with durations between 2 ms and 100 ms and a step count greater than 20 were extracted. For every batch of events, step sequences were padded with zeros to an equal length.

#### Model.

In pytorch (v2.6.0, CUDA 12.6), the BiLSTM classifier model was constructed as follows. The first chunk of the model is a bidirectional LSTM with an input dimension of 1 which takes in the sequence of steps one by one, hidden dimension N_H_, layer count N_L_, and internal drop out fraction D. The output of the BiLSTM chunk consists of the hidden states of the final layer, having a dimension of 2N_H_ (N_H_ for each direction). These two vectors were concatenated into a 1D vector. Another dropout layer was imposed on the concatenated vector with fraction D. Then, the 2N_H_ vector was passed to the linear (dense) classifier head, consisting of a layer with 2N_H_ input and N_H_ output, a ReLU function in between, and a final layer with N_H_ input and 5 output, producing the logits, where the element with maximum value is the identified class of protein.

#### Training.

The optimizer used here is AdamW, a variant of the Adam optimizer with weight decay to minimize overfitting during the training session. The loss function is Categorical Cross-entropy. Over multiple trials using constant learning rate and stepwise reduced learning rate, we found that stepwise reduction of learning rate by a factor of γ performed slightly better than constant learning rates across many tuning trials.

### Expression of P2 in *E. coli* for downstream lysate experiments

Plasmid encoding for P2 was transformed into NEB BL21 (DE3) cells following the 5 Minute heat shock transformation protocol. Next, cells were grown overnight at 37 °C on agar plates containing 100 μg/mL ampicillin. A single colony was inoculated into a flask with 50 mL of LB medium containing 100 μg/mL ampicillin in a 250 mL Erlenmeyer flask and grown at 37 °C while shaking at 220 rpm. The optical density of the medium was checked every 30 minutes until the culture reached an OD_600_ of 0.6, which is when P2 expression was induced with 1 mM IPTG and incubated at 37 °C while shaking at 220 rpm. During the 3-hour induction period, we collected two replicate 1 mL aliquots at six time points (0, 0.5, 1, 1.5, 2, and 3 hours) from the 50 mL culture. Aliquots were immediately placed on ice for 10 minutes and then stored in −80 °C. For each time point, one aliquot was used for Western Blot analysis and the other for our nanopore approach.

### Gompertz growth model fitting and estimated parameters

Growth trajectories of P2 expression in *E. coli* BL21(DE3) measured with both Western Blots and our nanopore assay were modeled using the Gompertz^98^ function:

$$y\left(t\right)=\text{Dexp}[-exp\frac{\mu_{max^{e}}}{D}(\lambda -t)+1]$$

where y(t) is the protein expression level at time t, D is the predicted plateau (maximum attainable expression level) under the experimental conditions, μ_max_ is the maximum specific expression rate (maximum slope at the inflection point), λ is the lag time parameter before rapid expression begins (after IPTG induction). Estimates for these fitting parameters for measurements obtained with our nanopore approach and Western blot are shown in the table below:

**Table:** 

Parameter	Western Blot	Nanopore
D	4,808 nM	5,362 nM
μ_max_	3,962 nM/hr	2,608 nM/hr
λ	0.225 h	0.341 h

## Extended Data

## Supplementary Material

Supplementary Files

This is a list of supplementary files associated with this preprint. Click to download.

- MakhamrehetalNBiotechappealedSI.pdf
- transtocis.mp4
- Reportingsummary.pdf
- cistotrans.mp4

## Figures and Tables

**Table 1:** Inclusion body isolation buffers for PBI analytes

Lysis Buffer	200 mM NaCl, 50 mM Tris-HCl pH 7.5, 5 mM EDTA, 100 μg/mL Lysozyme, 1% Triton-X 100, 1x Pierce protease inhibitor, 1 mM PMSF
Wash Buffer 1	200 mM NaCl, 20 mM Tris-HCl pH 7.5, 1 mM MgCl2, Benzonase nuclease
Wash Buffer 2	200 mM NaCl, 20 mM Tris-HCl pH 7.5, 2 mM EDTA

**Table 2:** IMAC buffers for purification of PBI analytes

Wash Buffer	200 mM NaCl, 20 mM Tris-HCl, 20 mM imidazole, pH 7.5, 6 M GdmCl
Elution Buffer	200 mM NaCl, 20 mM Tris-HCl, 500 mM imidazole, pH 7.5, 6 M GdmCl

## Data Availability

Original binary data files for MBP, each PBI analyte, and unlabeled mixtures are available on the Pennsieve Discover database (https://discover.pennsieve.io/datasets/454).

## Code Availability

All data preprocessing (filtering, parsing, and segmentation) were performed using the Ionique package (https://github.com/wanunulab/ionique). All code for analysis and figure generation was written in Python and is available as Jupyter notebooks in the nanopore-pbi-fingerprinting repository (https://github.com/wanunulab/nanopore-pbi-fingerprinting)
